# Identification of coding and non-coding mutational hotspots in cancer genomes

**DOI:** 10.1186/s12864-016-3420-9

**Published:** 2017-01-05

**Authors:** Scott W. Piraino, Simon J. Furney

**Affiliations:** 1 School of Biomolecular and Biomedical Science, Conway Institute of Biomolecular and Biomedical Research, University College Dublin, Dublin, Ireland; 2 School of Biomolecular and Biomedical Science, Conway Institute of Biomolecular and Biomedical Research, University College Dublin, Belfield, Dublin 4, Ireland

**Keywords:** Cancer genome sequencing, Non-coding mutations, Mutational hotspots

## Abstract

**Background:**

The identification of mutations that play a causal role in tumour development, so called “driver” mutations, is of critical importance for understanding how cancers form and how they might be treated. Several large cancer sequencing projects have identified genes that are recurrently mutated in cancer patients, suggesting a role in tumourigenesis. While the landscape of coding drivers has been extensively studied and many of the most prominent driver genes are well characterised, comparatively less is known about the role of mutations in the non-coding regions of the genome in cancer development. The continuing fall in genome sequencing costs has resulted in a concomitant increase in the number of cancer whole genome sequences being produced, facilitating systematic interrogation of both the coding and non-coding regions of cancer genomes.

**Results:**

To examine the mutational landscapes of tumour genomes we have developed a novel method to identify mutational hotspots in tumour genomes using both mutational data and information on evolutionary conservation. We have applied our methodology to over 1300 whole cancer genomes and show that it identifies prominent coding and non-coding regions that are known or highly suspected to play a role in cancer. Importantly, we applied our method to the entire genome, rather than relying on predefined annotations (*e.g.* promoter regions) and we highlight recurrently mutated regions that may have resulted from increased exposure to mutational processes rather than selection, some of which have been identified previously as targets of selection. Finally, we implicate several pan-cancer and cancer-specific candidate non-coding regions, which could be involved in tumourigenesis.

**Conclusions:**

We have developed a framework to identify mutational hotspots in cancer genomes, which is applicable to the entire genome. This framework identifies known and novel coding and non-coding mutional hotspots and can be used to differentiate candidate driver regions from likely passenger regions susceptible to somatic mutation.

**Electronic supplementary material:**

The online version of this article (doi:10.1186/s12864-016-3420-9) contains supplementary material, which is available to authorized users.

## Background

The characterisation of driver mutations in tumour genomes is a major component of cancer genomics research [1–3]. Cancer develops when somatic cells sustain genetic damage. Some mutations generated in this manner allow a cell and its progeny to survive and divide more rapidly, eventually generating a detectable tumour. However, a large fraction of mutations present in cancer genomes do not confer a detectable advantage to cells, therefore do not experience somatic selection and are termed passenger mutations. The mutations that do confer an advantage to cancerous cells are positively selected during tumour development, and are referred to as driver mutations [4]. Driver mutations are causally related to the development of individual cancers, so cataloging potential driver mutations is critical to understanding the mechanisms and dynamics of tumour development. Additionally, because driver mutations contribute to and sometimes are essential for the growth and survival of a tumour, the presence or absence of specific driver mutations are strong candidate biomarkers for personalized cancer therapies.

Driver mutations within the coding regions of the genome have been extensively characterized [4–8]. This has generally taken the form of large studies both within and across cancer types that have attempted to identify driver genes (genes that contain driver mutations). As a result of this work, several strategies have been developed that can be used to infer regions that are targets of positive somatic selection (putative driver regions) from the somatic mutations present in large sets of tumours. Positive selection is expected to increase the frequency with which a mutation is observed in sequencing experiments above the rate expected simply from mutational processes alone. As a result, recurrence of a mutation, or mutations within a given region of the genome relative to the mutation rate of that region is a signal of positive selection [6–9]. Driver mutations are also likely to be mutations that have strong functional effects. As a result, the functional consequence of a mutation can be an indication of the likelihood that a mutation or region has driver potential [10]. In the context of coding mutations for example, nonsynonymous mutations are *apriori* more likely to be driver mutations than synonymous mutations. Driver mutations often display a clustered pattern within driver regions across tumours, particularly in oncogenes [11, 12]. This can be the case when mutations in two separate tumours target the same functional site or domain, creating a clustered pattern where mutations tend to occur within the same region, and are mutually exclusive across individual tumours (i.e. only one mutation at the site per tumour).

Most efforts to characterize driver mutations have focused exclusively on coding regions of the genome, but recent examples of non-coding mutations that can contribute to tumourigenesis have sparked interest in the non-coding regions of the cancer genome [13]. For example, mutations in the promoter of the telomerase reverse transcriptase (*TERT*) gene have been identified as pan-cancer driver mutations that function by creation of a *de novo* transcription factor binding site upstream of *TERT*, resulting in TERT mRNA upregulation [14, 15]. *TERT* mutations occur recurrently at two nucleotides upstream of *TERT* in a mutually exclusive manner. Several studies have also conducted systematical screens of the non-coding regions of the genome for driver mutations [16–25]. These efforts have mainly focused on identifying recurrently mutated regions, but have also included other approaches. In the context of non-coding mutations, one potential strategy is to use various annotations to increase the priority given to certain types of mutations, similar to the use of annotations (*e.g.* PolyPhen, SIFT) for coding mutations. Examples of annotations that have been applied to non-coding mutations include information about motif disruption/creation [19, 21, 24] and human germline polymorphism frequency [19]. Other studies have correlated non-coding mutation status with mRNA expression [18, 21] and clinical data [21, 26].

These studies have predominantly focused on the subset of the non-coding genome that is most likely to be functional (*e.g*. promoter or regulatory regions). However, there may be driver regions that lie outside of currently known functional regions or in less well-documented and studied regions. As such, the aforementioned studies notwithstanding, the extent and significance of the contribution of non-coding mutations in cancer development has yet to be fully eludicated. This is in part due to the fact that we do not possess a clear appreciation of how to extricate the information from cancer genomes necessary to interpret the significance of non-coding mutations.

Therefore, in this study we sought to develop a novel method for the identification of mutational hotspots in cancer genomes that can be applied to prioritize putative non-coding driver regions in cancer. First, we aimed to develop a method that was applicable to entire genome, both coding and non-coding, rather than only a subset of regions. Second, we decided to incorporate information on evolution conservation in addition to mutation recurrence, and to determine what impact the inclusion of this information has on the regions identified. We developed a procedure for validating the performance of our scoring method that is based on the ability to identify known driver genes within coding regions. We also applied our method in a cancer type specific analysis to evaluate the possibility that some non-coding driver regions might be mutated in a cancer type specific manner.

## Results

We have developed a scoring method, described in detail below, that identifies regions of the genome that are more frequently mutated compared to flanking regions (recurrence score) and that have mutations at bases that are more highly conserved (conservation score). We have applied this method to 1349 whole cancer genomes from a variety of cancer types (Additional file 1: Table S1) for 50 bp windows spanning the entire human genome. Unlike previous efforts aimed at identifying non-coding driver mutations, which have usually focused on a limited set of non-coding regions (*e.g.* promoters, DNase I hypersensitive sites) we have applied our method in an unbiased manner to the entire genome, with the sole exception of regions where mappability is a concern. Here, we examine the characteristics and performance of our scores, as well as highlighting some promising candidate regions.

### Mutational processes in cancer genomes

Our objective was to identify regions of the non-coding genome that are under positive selection during tumourogenesis. We searched for regions of the genome that are recurrently somatically mutated in cancer, a signal of positive selection. Although recurrent mutation may be a result of selection, it may also result from mutational processes acting on cancer genomes. There is considerable heterogeneity in mutation rates between different regions of the genome [9] as well as between different tumours (Additional file 2: Figure S1). To discover regions that are mutated more than would be expected from underlying mutational processes, we implemented a score that normalized for the mutation rate in flanking regions. This method can account for mutational processes that are constant over large portions of the genome, but may falsely identify portions of the genome that are particularly susceptible to mutation within a focused region. Because of the possibility that such focal mutational processes might contaminate regions identified by our scoring method, we additionally sought to understand mutational processes acting on whole cancer genomes for the purpose of flagging regions that are potential false positives.

### Identification of putative hypermutated regions

**Fig. 1:**
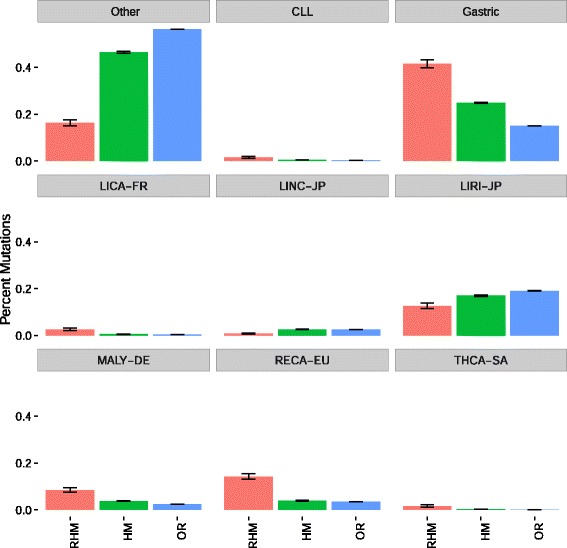
For each of three categories: recurrent and hyper mutated regions (RHM, red, 832 total mutations), non-recurrent hypermutated regions (HM, green, 20958 total mutations), and other regions (OR, blue, 10713694 total mutations), we show the percent of mutations within region that belong to different cancer types. Malignant lymphoma has a disproportionate share of hypermutated regions, suggesting that our method of identifying hypermutated regions is capturing some regions that are targets of somatic hypermutation in this cohort. We define a region to be hypermutated when it has > 1.2 mutations per tumour, and to be recurrently mutated when it has a recurrence score greater than 10

**Fig. 2:**
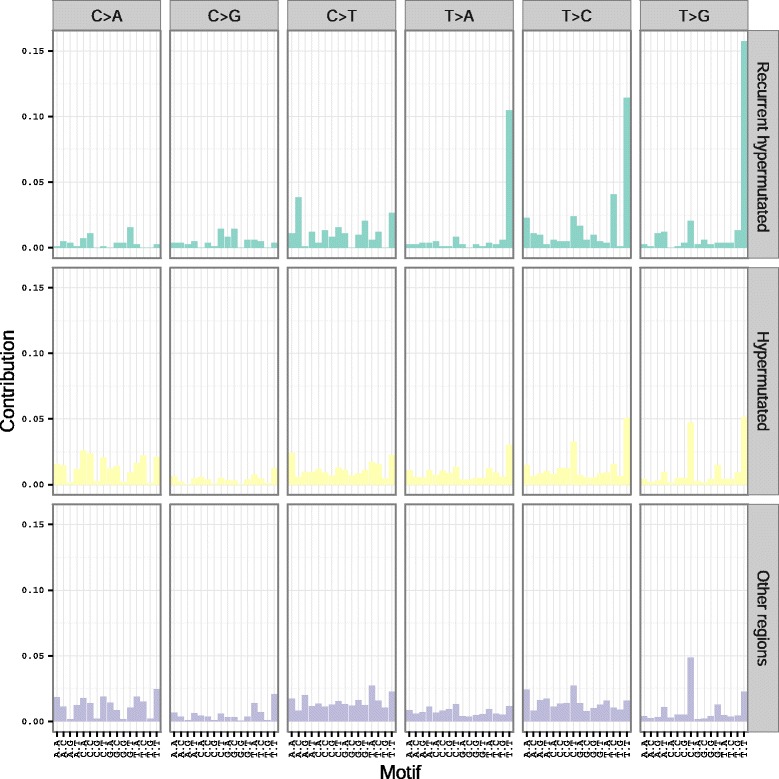
Observed mutational spectra within recurrent hypermutated, non-recurrent hypermutated, and non-hypermutated regions. Each column represents a particular category of mutation, defined by the base change, as well as the bases that flank the mutated nucleotide, both 5’ and 3’. The height of each bar is proportional to the frequency of the mutational category within each region type

We reasoned that regions of the genome with unusually high exposure to mutational processes would be expected to have a consistently elevated likelihood of mutation, whereas selection is expected to diminish once a driver has already been mutated. For example, gain of function mutations in oncogenes generally only need to occur once to confer driver activity, and often display mutual exclusivity with other mutations that have the same effects or that target the same pathway. Tumour suppressor genes are an exception, where two mutations may be required to confer driver activity. Thus, regions that are susceptible to mutation are more likely to sustain repeated mutations within the same region in the same tumour, while regions that are recurrently mutated due to selection are more likely to be mutated only once per tumour. In order to identify regions that may be recurrently mutated due to mutational processes rather than selection, we calculated the average number of mutations per patient for each region under consideration. We considered a region to be potentially hypermutated when the region had an average of 1.2 mutations per mutated patient or greater. We examined the prevalence of mutations within these putative hypermutated regions across tumour types. Several tumour types have an excess of mutations from hypermutated regions (Fig. 1) such as lymphomas (“MALY-DE”) and renal cancers (“RECA-EU”). Several of the regions that we have identified as being hypermutated by this method lie in promoter regions and are primarily mutated in lymphoma, potentially suggesting that these regions are targets of somatic hypermutation rather than selection. Furthermore, some of these regions such as the promoter regions of *BCL2* and *MYC* have been identified as putative targets of selection in a previous analysis [26]. Analysis of mutational signatures within the putatively hypermutated regions that we identified did not identify any specific mutation process that could explain the pattern of base substitutions in these regions (Fig. 2), although it is possible that this mutational pattern is partially due to a process identified in CLL and lymphoma that is implicated with AID induced somatic hypermutation [27]. To evaluate the possible sensitivity of our method for identifying hypermutated regions to the specific threshold we use, we compared the classification of regions at a threshold of 1.2 with several other thresholds. For all values, > 97% of regions received the same designation (hypermutated vs non-hypermutated) when compared to the 1.2 threshold. We therefore use the > 1.2 threshold throughout the rest of our analysis.

### Mutational processes at CTCF binding sites

**Fig. 3:**
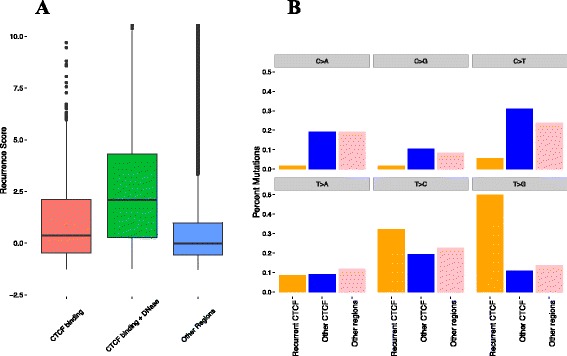
(**a**) CTCF binding sites that overlap (green) and do not overlap (red) DNase I hypersensitive sites show a higher recurrence score compared to non-CTCF binding regions (blue); (**b**) We classified mutations as coming from recurrent CTCF binding sites (orange), non-recurrent CTCF binding sites (blue) and non-CTCF binding sites (pink). For each of these three categories, we give percentages indicating how many mutations from each category exhibit each of the six possible base changes. We define a CTCF binding site as recurrent when it has a recurrence score greater than 10

In addition to the putatively hypermutated regions that we identified, we also observed that many recurrently mutated regions overlap regions with ChIP-seq evidence of CTCF binding (Fig. 3a, CTCF binding vs other regions *p* = 3.8 x 10^−18^, CTCF DNase I hypersensitive vs other regions *p* = 2.08 x 10^−263^, CTCF binding vs CTCF DNase I hypersensitive *p* = 1.24 x 10^−46^). A recent analysis also identified an association between CTCF binding and recurrent mutation [20] potentially suggesting selection of these mutations, while other evidence from colorectal cancer by Katainen *et al.* suggests that CTCF binding sites may be subject to a unique mutational process which displays an excess of T > G (A > C) and T > C (A > G) mutations [28]. To discern whether the observed recurrence at CTCF binding sites in our dataset could result from a mutational process rather than selection, we compared the mutations at CTCF binding sites with the signature observed in Katainen *et al.* [28]. While CTCF binding sites in general do not show a signature similar to the one in [28] CTCF binding sites that we also identified as recurrent in our analysis display an excess of T > G and T > C mutations (Fig. 3b). When we examined specific recurrently mutated CTCF binding site that was also identified in [28] we found that the same bases within the binding site were recurrently mutated (Additional file 2: Figure S2). This suggests that the recurrently mutated CTCF binding sites identified by our analysis are likely the result of the same process implicated in Katainen *et al.* [28]. CTCF binding sites that additionally have overlapping evidence of DNase I hypersensitivity in encode data display increased recurrence scores, consistent with the explanation that these mutations are the result of a mutational process related to DNA repair [29]. Many of the CTCF mutations in our sample come from a set of gastric cancer genomes, a cancer type not previously included by Katainen *et al.* Our analysis thus extends these observed patterns to this cancer type. Recent analyses have shown that transcription factor bound regions of the genome are subject to unique mutational processes and these mutations often preferentially target certain bases (e.g. G/C bases) [29, 30]. Our recurrence score correlates weakly with GC context (rank correlation 0.113) perhaps due to coding driver genes having high GC% (Additional file 2: Figure S3). Regions with recurrence score > 10 have comparable GC% to regions with score < 10 (Wilcoxon rank sum *p*-value = 0.81).

### Pan-cancer prioritisation of non-coding mutations

**Fig. 4:**
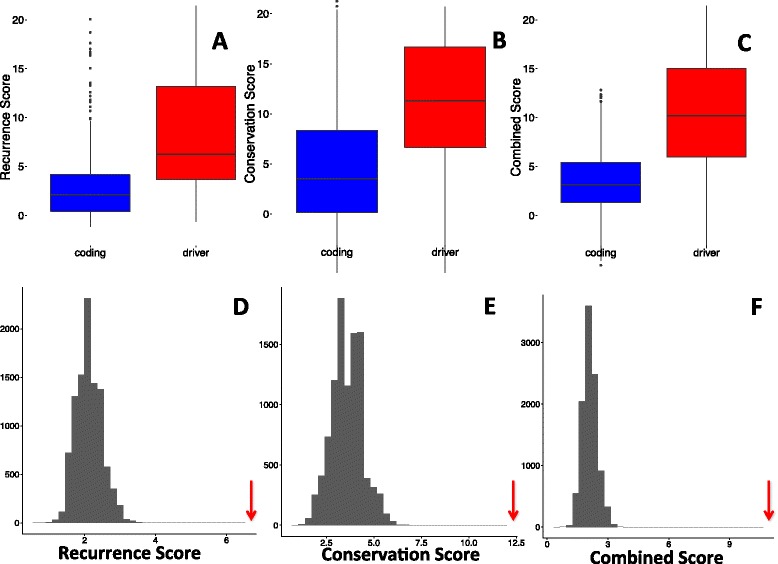
For exonic regions, known driver genes score significantly higher in terms of recurrence (**a**, **d**) conservation (**b**, **e**) and combined scores (**c**, **f**). We also compare the observed medians scores for drivers (red arrows) to median scores generated by resampling non-driver regions (grey bars, **d** - **e**)

Having identified CTCF binding sites and regions with >1.2 mutations per tumour as regions that might be enriched for false positives, we next sought to identify regions that were likely to be under selection. We validated our prioritisation scores by considering exonic regions within our sample, because many large analyses have already identified known driver genes in protein coding regions. Our recurrence score (*p* = 3.8 x 10^−27^), conservation score (*p* = 1.32 x 10^−19^), and combined score (*p* = 3.22 x 10^−30^) were able to discriminate known driver genes within the set of all exonic regions (Fig. 4 a-c), suggesting that our method has reasonable effectiveness within this subset of the genome, despite the fact that we did not take advantage of annotations that are available for coding mutations (*e.g.* non-synonymous vs synonymous mutations). We confirmed this by direct comparison of scores between driver and non-driver regions, as well as by simulation. To compare the known driver regions to a set of non-drivers of equal size, we resampled the non-driver exonic regions 10,000 times for each score, and compared the median score of the sampled non-drivers to the observed median of the known drivers. For all three scores, none of the 10,000 samples exceeded the median driver score (Fig. 4 d-f). Several of the top scoring coding regions overlap well-known driver genes such as *TP53* and *KRAS*. To investigate whether the inclusion of coding sequence within flanking regions had an impact on the regions identified, we also rescored each candidate region, this time excluding coding regions from the calculation of the flanking mutation rate. The regions identified were largely similar, with 94% of top regions in common between the two scoring methods. In order to assess whether the mutational counts are dominated by hypermutated samples, we recalculated the number of mutations in each 50 bp window, excluding samples that are two standard deviations above the mean number of mutations. These counts are highly correlated (*r* = 0.88, *p* < 0.0001) and this correlation is maintained when considering only regions that have greater than 5 mutations in the full dataset (*r* = 0.937, *p* < 0.00001).

**Fig. 5:**
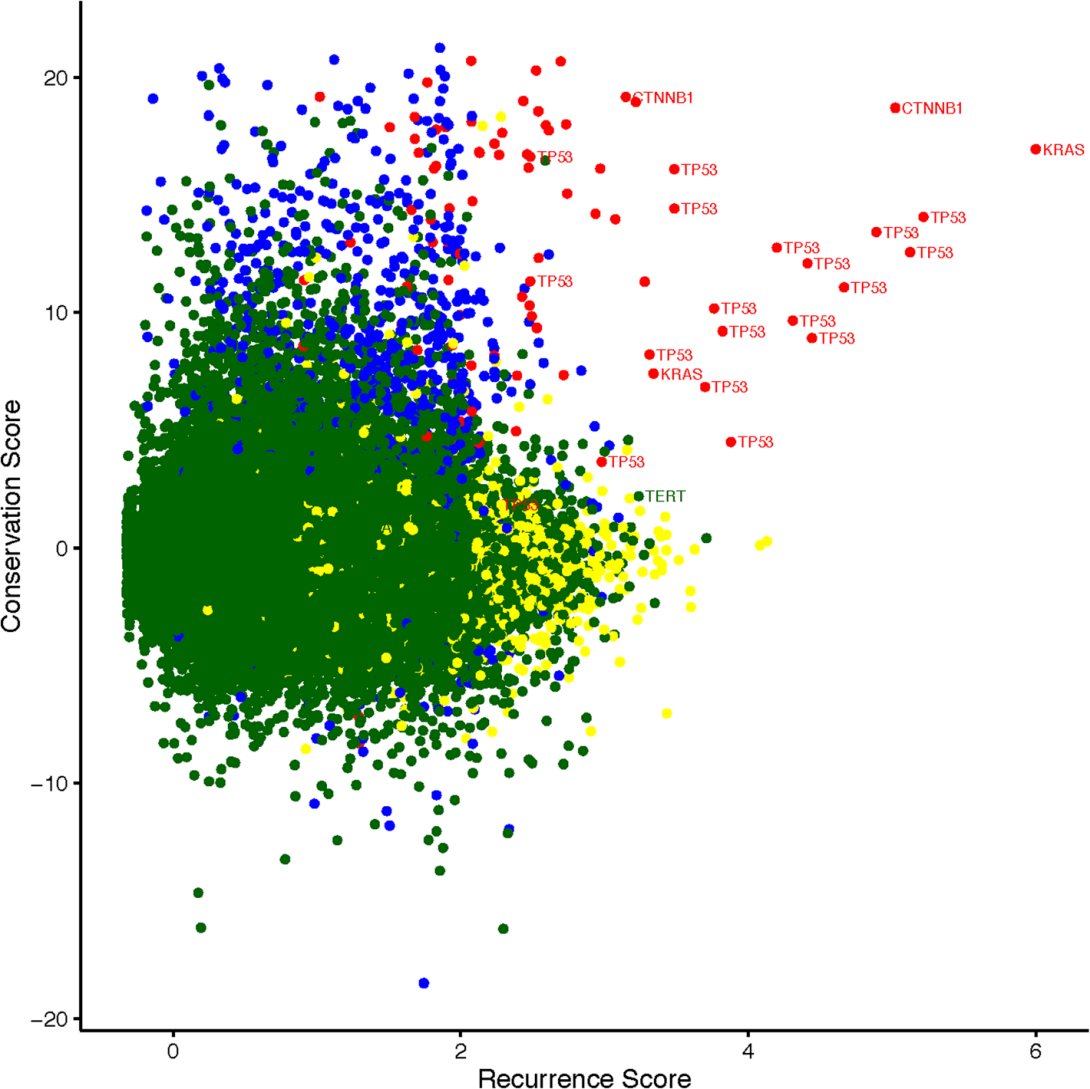
Scatterplot of all regions mutated in more than two patients with conservation score on the vertical axis and Log (recurrence score + 2) on the horizontal axis. The points are colored based on a classification of each region into one of four categories: coding, non-driver regions (blue), coding driver regions (red), non-coding, hypermutated regions (yellow), and non-coding non-hypermutated regions (green). Several known driver regions are also labelled

In addition to identifying known coding drivers, we also identified recurrently mutated non-coding regions, including both previously identified regions as well as novel regions (Fig. 5; Tables 1, 2, 3 and 4). We identified *TERT* (Additional file 2: Figure S4) and *PLEKHS1* (Additional file 2: Figure S5) promoters as being recurrently mutated, consistent with previous analyses [21]. *TERT* appears in the top 50 regions genome-wide by recurrence (Table 1) but not when ranked by the combined score (Table 3). One explanation for this is that in a genome-wide context, adding conservation will tend prioritise coding regions more highly, given the higher conservation of coding compared to non-coding regions. In support of this interpretation, Table 3 appears to be enriched for coding drivers relative to Table 1, while comparison of the top ten non-coding, non-hypermutated regions based on recurrence (Table 2) and combined score (Table 4) are highly similar. Despite the similarity of these lists, adding conservation does prioritise some interesting regions, including an intronic region that shows high conservation, as well as a conserved region of a miRNA. We discuss several candidate regions in more detail in the next section.

**Table 1:** Top 50 regions in terms of recurrence score identified by our method. We give the position of the region, number of genomes that are mutated within the region, the recurrence score, and a classification of the region based annotations and our method of identifying hypermutated regions. We also manually annotated each region by viewing in the UCSC genome browser

Rank	Chr	Start	End	Mutated samples	Score	Automated annotation	Manual annotation
1	chr12	25398250	25398300	256	399.9	Driver	*KRAS* exon
2	chr17	7577100	7577150	68	182.1	Driver	*TP53* exon
3	chr17	7577500	7577550	62	165.7	Driver	*TP53* exon
4	chr3	41266100	41266150	65	149.3	Driver	*CTNNB1* exon
5	chr17	7578400	7578450	50	130.6	Driver	*TP53* exon
6	chr17	7577550	7577600	41	103.9	Driver	*TP53* exon
7	chr17	7578200	7578250	32	82.8	Driver	*TP53* exon
8	chr17	7578250	7578300	31	80.1	Driver	*TP53* exon
9	chr17	7577050	7577100	29	72.2	Driver	*TP53* exon
10	chr17	7578500	7578550	26	64.4	driver	*TP53* exon
11	chr10	96652800	96652850	14	60.0	hotspot	non-coding
12	chr12	6899300	6899350	3	57.1	hotspot	*CD4* intron
13	chr17	7574000	7574050	19	46.2	driver	*TP53* exon
14	chr17	7578450	7578500	18	43.5	driver	*TP53* exon
15	chr17	7578350	7578400	17	40.9	driver	*TP53* exon
16	chr3	195892250	195892300	18	38.7	non-coding	non-coding
17	chr17	7577000	7577050	14	38.3	driver	*TP53* exon
18	chr12	64749950	64750000	7	35.5	hotspot	*C12orf56* intron
19	chr13	50016900	50016950	8	34.5	hotspot	*CAB39L* intron
20	chr11	63881800	63881850	9	34.4	hotspot	*FLRT1* intron
21	chr15	64857000	64857050	9	31.6	hotspot	*ZNF609* intron
22	chr17	7578150	7578200	13	30.6	driver	*TP53* exon
23	chr17	7578550	7578600	13	30.5	driver	*TP53* splice site
24	chr16	88383450	88383500	7	28.9	hotspot	Non-coding / TF binding
25	chr14	24895100	24895150	11	28.8	hotspot	Non-coding / TF binding
26	chr17	79389900	79389950	9	28.8	hotspot	*BAHCC1* intron
27	chr17	17424850	17424900	7	28.5	hotspot	*PEMT* intron
28	chr22	46697350	46697400	5	27.8	hotspot	*GTSE1* intron
29	chr8	30717550	30717600	7	27.8	hotspot	*TEX15* exon-intron border
30	chr7	76949650	76949700	6	27.6	hotspot	*GSAP* intron
31	chr14	74239050	74239100	8	27.2	hotspot	*ELMSAN1* intron
32	chr4	819750	819800	6	27.0	hotspot	*CPLX1* intron
33	chr16	81908550	81908600	7	26.4	hotspot	*PLCG2* intron
34	chr4	39684550	39684600	10	26.4	non-coding	non-coding
35	chr22	39962000	39962050	6	26.2	hotspot	non-coding
36	chr12	25380250	25380300	20	26.1	driver	*KRAS* exon
37	chr3	43746400	43746450	11	25.4	non-coding	*ABHD5* intron
38	chr17	7579300	7579350	10	25.4	driver	*TP53* exon
39	chr9	21971100	21971150	12	24.5	driver	*CDKN2A* exon
40	chr8	9921850	9921900	12	24.3	non-coding	*MRSA* intron
41	chr11	70764100	70764150	6	24.1	hotspot	*SHANK2* intron
42	chr19	12597300	12597350	9	23.8	hotspot	*ZNF709* intron
43	chr17	49455750	49455800	10	23.6	hotspot	non-coding
44	chr5	1295200	1295250	14	23.4	non-coding	*TERT* promoter
45	chr7	151591800	151591850	6	23.2	hotspot	non-coding
46	chr21	44524450	44524500	9	22.9	driver	*U2AF1* exon
47	chr1	45914900	45914950	7	22.7	hotspot	*TESK2* intron
48	chr8	29901300	29901350	9	22.4	non-coding	non-coding
49	chr7	606050	606100	7	22.0	hotspot	*PRKAR1B* intron
50	chr2	49173750	49173800	27	22.0	non-coding	CTCF binding

**Table 2:** Top ten non-coding, non-hypermutated regions in terms of recurrence score

rank	chr	start	end	samples mutated	score	manual annotation
1	chr3	195892250	195892300	18	38.7	non-coding
2	chr4	39684550	39684600	10	26.4	non-coding
3	chr3	43746400	43746450	11	25.4	*ABHD5* intron
4	chr8	9921850	9921900	12	24.3	*MSRA* intron
5	chr5	1295200	1295250	14	23.4	*TERT* promoter
6	chr8	29901300	29901350	9	22.4	non-coding
7	chr2	49173750	49173800	27	22.0	CTCF binding
8	chr8	70576150	70576200	21	21.8	CTCF binding
9	chr19	893450	893500	9	21.6	*MED16* promoter
10	chr2	47359300	47359350	8	21.0	*C2orf61* intron

**Table 3:** Top 50 regions in terms of combined score identified by our method. We give the position of the region, number of genomes that are mutated within the region, the combined score, and a classification of the region based annotations and our method of identifying hypermutated regions. We also manually annotated each region by viewing in the UCSC genome browser

rank	chr	Start	End	Mutated samples	Score	Automated annotation	Manual annotation
1	chr12	25398250	25398300	256	208.4	driver	*KRAS* exon
2	chr17	7577100	7577150	68	98.1	driver	*TP53* exon
3	chr17	7577500	7577550	62	89.1	driver	*TP53* exon
4	chr3	41266100	41266150	65	84.0	driver	*CTNNB1* exon
5	chr17	7578400	7578450	50	72.0	driver	*TP53* exon
6	chr17	7577550	7577600	41	57.5	driver	*TP53* exon
7	chr17	7578250	7578300	31	46.1	driver	*TP53* exon
8	chr17	7578200	7578250	32	45.8	driver	*TP53* exon
9	chr17	7577050	7577100	29	40.9	driver	*TP53* exon
10	chr17	7578500	7578550	26	38.6	driver	*TP53* exon
11	chr10	96652800	96652850	14	30.1	hotspot	Non-coding
12	chr12	6899300	6899350	3	28.6	hotspot	*CD4* intron
13	chr17	7578450	7578500	18	26.4	driver	*TP53* exon
14	chr17	7578350	7578400	17	25.5	driver	*TP53* exon
15	chr17	7574000	7574050	19	25.4	driver	*TP53* exon
16	chr17	7578550	7578600	13	23.3	driver	*TP53* exon
17	chr17	7577000	7577050	14	22.6	driver	*TP53* exon
18	chr17	7578150	7578200	13	22.5	driver	*TP53* exon
19	chr21	44524450	44524500	9	20.9	driver	*TP53* exon
20	chr3	41266050	41266100	10	20.2	driver	*CTNNB1* exon
21	chr3	195892250	195892300	18	19.5	non-coding	Non-coding
22	chr9	21971100	21971150	12	17.9	driver	*CDKN2A* exon
23	chr12	64749950	64750000	7	17.7	hotspot	*C12orf56* intron
24	chr17	7579300	7579350	10	16.8	driver	*TP53* exon
25	chr2	198266800	198266850	9	16.8	driver	*SF3B1* exon
26	chr12	25380250	25380300	20	16.8	driver	*KRAS* exon
27	chr18	48591900	48591950	11	16.8	driver	*SMAD4* exon
28	chr3	178936050	178936100	9	16.7	driver	*PIK3CA* exon
29	chr11	63881800	63881850	9	16.3	hotspot	*FLRT1* intron
30	chr13	50016900	50016950	8	16.0	hotspot	*CAB39L* intron
31	chr19	11134250	11134300	6	15.7	driver	*SMARCA4* exon
32	chr15	64857000	64857050	9	15.5	hotspot	*ZNF609* intron
33	chr20	57484400	57484450	13	15.5	driver	*GNAS* exon
34	chr16	3786700	3786750	5	15.4	driver	*CREBBP* exon
35	chr17	17424850	17424900	7	14.9	hotspot	*PEMT* intron
36	chr14	24895100	24895150	11	14.7	hotspot	Non-coding / TF binding
37	chr18	48575150	48575200	7	14.7	driver	*SMAD4* exon
38	chr18	48604750	48604800	7	14.6	driver	*SMAD4* exon
39	chr19	11132500	11132550	5	14.6	driver	*SMARCA4* exon
40	chr17	79389900	79389950	9	14.3	hotspot	*BAHCC1* exon
41	chr18	48591800	48591850	8	14.2	driver	*SMAD4* exon
42	chr3	178952050	178952100	7	14.2	driver	*PIK3CA* exon
43	chr7	76949650	76949700	6	14.0	hotspot	*GSAP* intron
44	chr14	74239050	74239100	8	13.9	hotspot	*ELMSAN1* intron
45	chr17	56408600	56408650	5	13.9	non-coding	*MIR142* non-coding
46	chr22	46697350	46697400	5	13.6	hotspot	*GTSE1* intron
47	chr8	30717550	30717600	7	13.4	hotspot	*TEX15* exon-intron border
48	chr10	89692900	89692950	3	13.3	driver	*PTEN* exon
49	chr17	7577600	7577650	5	13.3	driver	*TP53* splice site
50	chr4	819750	819800	6	13.2	hotspot	*CPLX1* intron

**Table 4:** Top ten non-coding, non-hypermutated regions in terms of combined score

rank	chr	start	end	samples mutated	score	manual annotation
1	chr3	195892250	195892300	18	38.7	non-coding
2	chr4	39684550	39684600	10	26.4	non-coding
3	chr3	43746400	43746450	11	25.4	*ABHD5* intron
4	chr8	9921850	9921900	12	24.3	*MSRA* intron
5	chr5	1295200	1295250	14	23.4	*TERT* promoter
6	chr8	29901300	29901350	9	22.4	non-coding
7	chr2	49173750	49173800	27	22.0	CTCF binding
8	chr19	893450	893500	9	21.6	*MED16* promoter
9	chr6	142706200	142706250	9	18.0	*GPR126* intron
10	chr17	56408600	56408650	5	11.3	*MIR142*

### Novel recurrent non-coding mutations

Our method has highlighted several novel non-coding regions that may be selected for in cancer. Many highly recurrent regions are either known coding drivers or are regions that we have identified as hypermutated. Although a region can be both hypermutated and selected, we focus on highlighting regions that are less likely to hypermutated. To demonstrate the types of novel regions identified by our analysis, we examined several regions that scored among the top regions in terms of both recurrence and conservation scores in our pan-cancer analysis.

The first region that we examined lies between the protein-coding gene *MED16* and the small nuclear RNA *RNU6-2* (Additional file 2: Figure S6). This regions lies within a DNase I hypersensitivity site and shows heavy transcription factor binding, suggestive of promoter activity or some other regulatory function. Each mutation within the region lies within a conserved sub-region of the window. No mutations fall within the unconserved regions surrounding this sub-region or within the nearby RNA gene, despite the fact that these latter regions make up the majority of the window. Driver mutations often displaying clustering within specific functional regions. The pattern observed in this region, with mutations clustered within a single conserved element, is potentially suggestive of driver activity. Given the evidence for transcription factor binding in this region, one possibility is that this conserved sub-region is a motif associated with protein binding. Although mutations at this locus are focused within this conserved sub-region, the mutations are spread throughout the sub-region, not focused at any single nucleotide, and do not always show consistent base changes in the cases where the mutations do occur at the same nucleotide. Assuming that these mutations are in fact targeting some kind of binding motif, the relatively even distribution of mutations without consistent base changes possibly suggests that these mutations are disrupting a binding motif as opposed to a creating a novel motif. To assess the possibility that these mutations may alter protein-binding motifs at the site, we searched the reference sequence of the mutated region for possible matches with known motifs. We identified matches with the transcription factors FOXL1, NKX3-1, and MEF2A. We also searched for matches when the reference sequence is replaced with several of the mutants observed in our dataset. In the case of MEF2A both mutations we tested reduced the maximum similarity score from 13.7 to 5.7 and 0.92, suggesting that the mutations observed in this region may be disruptive to this motif (Additional file 2: Figure S7).

The second region that we highlight is deep within the intron of the gene *GPR126* (Additional file 2: Figure S8). This region shows high levels of conservation, and the mutations observed is region occur exclusively at two base positions. All mutations within this region are entirely mutually exclusive, and there are no other mutations within this region other than at these two positions. This pattern of mutation is similar to that initially observed at mutations in the *TERT* promoter, and is suggestive of driver activity. These mutations also occur at the same positions within a motif (GAAC) as mutations in the *PLEKHS1* promoter, potentially suggesting a common process is occurring at these two loci. These mutations lie far from any exon-intron boundaries, ruling out the possibility that they affect donor or acceptor sites. This region overlaps a DNase I hypersensitive site, potentially suggesting that this region contains on intronic regulatory elements. We identified motifs matching the transcription factors FOXL1, POU2F2, FOXA1, and FOXP2 overlapping this region. We did not notice a consistent pattern in the effects of the observed mutations on motif occurrence.

We additionally identified recurrent mutations at highly conserved positions overlapping the miRNA *MIR142* (Additional file 2: Figure S9). These mutations are spread throughout the region, and occur exclusively in lymphoma samples, suggesting that this region may be a target of somatic hypermutation. Puente *et al.* also identified recurrent mutations near *MIR142* in CLL, which they attribute to somatic hypermutation [22]. Despite the fact that this region may be a target of hypermutation rather than selection, the appearance of this region within the top ten non-coding, non-hypermutated regions in terms of combined score (Table 4) but not recurrence score (Table 2) suggests that conservation can highlight regions that are highly conserved but have lower recurrence. All but one of the mutations observed in our dataset overlap the mature microRNA hsa-miR-142-5p based on the miRBase [31] sequence (Additional file 2: Figure S10), suggesting that these mutations may have an impact of the ability of the mircoRNA to bind target mRNAs. This creates the possibility that this region is a target of both hypermutation and selection. As a result, it may be useful to use both scores separately to nominate regions with different characteristics. Finally, we highlight a recurrently mutated region in an intron in the gene *MSRA* (Additional file 2: Figure S11). Similar to several of the other regions highlighted, this region is mutated predominantly at two base positions, which in this case occur at neighbouring positions. We additionally identified motifs that are potential matches for transcription factors SOX9 and SRY overlapping this region. We did not notice a consistent pattern in the effects of the observed mutations on motif occurrence.

### Cancer type specific analysis

**Fig. 6:**
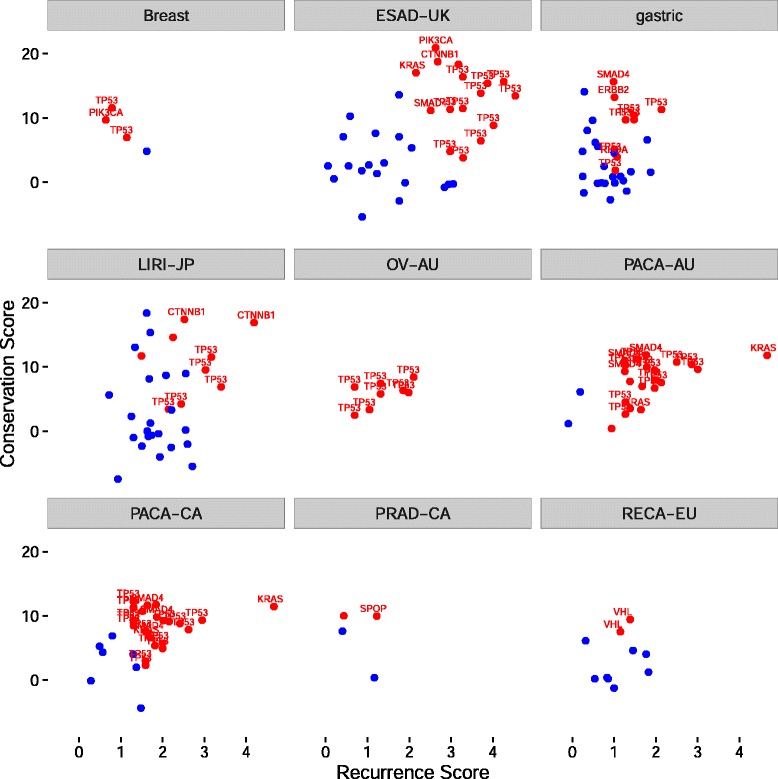
Scatterplots of exonic regions with three or more patients mutated within each cancer type. For each scatterplot, we plot regions mutated in three or more samples from a cancer type based on scores calculated only within each cancer type. Regions overlapping known driver genes are depicted in red, while other coding regions are depicted in blue. Several known driver genes are labeled in each plot

So far, we have focused on regions that are mutated in multiple cancer types. To investigate if some non-coding driver mutations are mutated primarily in one or a few cancer types only, we applied our scoring method independently to each cancer type in the dataset with more than 75 whole genomes. Consistent with our pan-cancer analysis, when we applied our method to the exonic regions of specific cancer types, we again identified many known cancer genes (Fig. 6). Several of the genes that we identified are particularly prominent in cancer types in which they are known to be highly mutated, such as *VHL* in renal cancer, *PIK3CA* in breast cancer, *TP53* in ovarian cancer, *SMAD4* in esophageal and gastric cancer, and *KRAS* in pancreatic cancer.

### Cancer type specific non-coding mutations

In addition to the regions identified in our pan-cancer analysis, we also identified non-coding regions that are recurrently mutated in individual cancer types (Additional file 1: Tables S2 and S3). We identified recurrent mutations within an intron of the *PRIM2* gene (Additional file 2: Figure S12) specifically in renal cancer. These mutations occurred at two bases in a mutually exclusive manner, and exclusively in renal cancer samples. We identified motifs matching the transcription factors FOXL1, BRCA1, FOXH1, FOXP1, PRDM1, TCF7L2, ZNF236, IRF1, STAT3, and FOXP2 overlapping this region. Two mutant sequences we tested had maximum scores of 11.1 compared to −0.8 for matches to FOXP2 (Additional file 2: Figure S13). We also identified recurrent mutations within an intron of *RAD51B* in several breast cancer samples (Additional file 2: Figure S14). *RAD51B* is a DNA repair gene involved in homologous recombination [32]. We identified motifs matching the transcription factors FOXC1, MZF1_5-13, MAFF, MAFK, EGR1, ESR2, GATA2, GATA3, and THAP1 overlapping this region. We did not notice a consistent pattern in the effects of the observed mutations on motif occurrence. Given the importance of this repair pathway in breast cancer, this region may warrant further study in this cancer type. Within the regions prioritised by the combined score, we also identified several extremely highly conserved regions that are recurrently mutated in the LIRI-JP cohort (liver cancer), including non-coding regions of the genes *BCL11A*, *BCL6*, and *PAX5* (Additional file 1: Table S3).

## Discussion

As is the case in the analysis of coding mutations, we have found that mutational heterogeneity is a critical factor that impacts the identification of non-coding driver regions in cancer. Our initial analysis revealed that several promising candidate regions, some of which have been suggested in the literature as potential driver regions, may actually be recurrently mutated primarily due to focal mutational processes rather than selection. We have found potential evidence of an AID associated somatic hypermutation signature as well as a recenty identified process which targets CTCF binding sites [28] as prominent local mutational processes. In addition, we have proposed methods for identifying and filtering out these putatively hypermutated regions, allowing greater focus on regions for which we believe the evidence favouring positive selection is stronger. Using the exome to validate our scoring method, we showed that all three scores can differentiate known drivers from other coding regions. We also identified several known driver genes that display a mutation pattern across cancer types consistent with expectations.

In addition to using recurrence as previous studies have, we included conservation as part of the prioritization scores. We have shown that the conservation score can separate known coding drivers from non-drivers. Conservation may also be useful in the analysis of non-coding mutations, both to increase confidence that recurrent non-coding mutations have the potential to impact function, as well as to highlight non-coding regions that may have lower recurrence but driver potential due to higher conservation. The combined score also appears to outperform the recurrence score alone in terms of distinguishing known driver regions from other exonic regions, suggesting that conservation provides valuable information in addition to recurrence, although this may be more difficult to interpret within the context of non-coding mutations, given that non-coding regions are generally less well conserved as a whole compared to coding regions. The generally low conservation observed in non-coding regions sugggests that functional non-coding mutations might not necessarily always occur at conserved positions. Thus, it is useful to consider recurrent mutations, even if they are not at highly conserved positions. Using a measure such as the combined score may also highlight regions that have moderate recurrence but which are highly conserved. These regions would be good candidates for more “hill-like” drivers [8]. As a result, we believe that using both recurrence and a combined score that incorporates recurrence and conservation to prioritise regions that may have different properties is a promising strategy. It is also worth noting that more complex ways of combining these scores might yield additional benefits. We have averaged the scores, after normalizing to make the scores roughly comparable, but other transformations might also produce insights.

Within these genomes, we also identified several novel recurrently mutated regions. In addition to the novel recurrent regions we identified in a pan-cancer analysis, we also identifed several novel non-coding regions that appear to be cancer type specific, some of which have high frequencies in the cancer types in which they occur. These regions, as well as other regions that score highly within our framework, may be good targets for future analyses of non-coding somatic mutations in cancer. Although the methods used here can not definitively establish a mutation as a driver, further investigation of non-coding mutations using these and other methods may reveal new non-coding driver mutations. These drivers may have important implications for cancer therapy if they are directly targetable by drugs or involved in the regulation of pathways that are targetable. Non-coding mutations such as *TERT* promoter mutations [33] have been associated with clinical outcomes, as have mutational processes in cancer [34–36]. We have highlighted regions that have an excess of mutations in cancer genomes. These regions may lead to important insights that may have clinical implications if they are either under selection or indicative of underlying mutational processes.

## Conclusions

We have developed a novel method for the identification of putative driver regions in cancer, which is applicable to both coding and non-coding regions. We have shown that this method performs well at identifying prominent coding and non-coding regions that are known or highly suspected to play a role in cancer. Unlike previous attempts to identify recurrently mutated non-coding regions, we apply our method to the entire genome to identify novel non-coding regions mutational hotspots. We also highlight recurrently mutated regions that may have resulted from increased exposure to mutational process rather than selection, some of which have been identified previously as targets of selection.

## Methods

In order to identify recurrently mutated non-coding regions that are potential targets of somatic selection during the development of cancer, we devised a scoring system to prioritise regions of the genome based on signatures that are indicative of selection. In the context of coding mutations, driver genes are known to be recurrently mutated above background mutation rates and also show a pattern of enrichment for functional mutations (e.g. stop-gain, non-synonymous) compared to mutations that are less likely to be function (e.g. synonymous mutations). Applying similar principles to non-coding regions, we developed two scores, one that is designed to detect regions that are recurrently mutated, and a second designed to detect regions that have mutations at conserved bases, working on the hypothesis that conserved positions are more likely to be functional. We then applied these scores, as well as a combined score, to a set of over 1300 cancer whole genomes.

### Whole genome mutation data

We assembled a set of pre-called somatic mutations from three sources: release 18 of ICGC [37], data from Alexandrov *et al.* [27], and the supplemental materials of Wang *et al.* [38]. Some of these sources contain data from both whole exome and whole genome sequencing. We only analyzed mutations annotated as coming from whole genome sequencing. To avoid the possibility of duplicated samples, in cases where the same tumour type was included in ICGC and the data from Alexandrov *et al.* we included data from only one source. The distribution of samples across tumour types and data sources is summarized in Additional file 1: Table S1. After filtering out samples lacking sufficient numbers of mutations, we were left with a total of 1349 samples for our final analysis.

### Annotation data

We used the UCSC genome browser [39, 40] to obtain various annotation files, including dbSNP and COSMIC variants, information on gene models, conservation, mappability, and epigenetic data.

### Software

We processed genomic data using bedtools v2.25.0 [41] and conducted statistical analysis and data manipulation in R 3.2.3 [42].

### Processing mutation data

We annotated all data to human reference genome version hg19. Preliminary analysis revealed several frequent mutations that overlap known germline SNPs, suggestive of the possibility that these mutations are not truly somatic. We removed from consideration mutations that occur at the same genomic coordinate as a known dbSNP entry, unless that genomic position was also annotated as mutated in COSMIC (cancer.sanger.ac.uk) [5]. After filtering out known dbSNP entries, we also excluded tumour samples with fewer than 1000 total mutations from further analysis. For dbSNP variants, we used build 142 of dbSNP. dbSNP and COSMIC variant locations were obtained in bed format from the UCSC Table Browser [39].

### Annotating and filtering genomic regions

We divided the reference hg19 genome into 50 bp, non-overlapping windows using the bedtools makewindows command. We mapped mutations to each window, and calculated the mean 100-way PhyloP score as well as the mean 35 bp uniqueness (a measure of sequence mappability) across mutations that fell within the window. We excluded from further consideration any window that had a mean mappability of its overlapping mutations that was less than 0.5, as well as any window that was mutated in fewer than 3 patients (because these regions lack sufficient mutations to be considered recurrent).

### Calculation of recurrence score

For each region that met our filtering criteria (candidate regions), we calculated a recurrence score representing the level of enrichment of the region with mutations compared to the mutation rate within the region of the genome flanking the region under consideration. For each candidate region, we formed a flanking region, which included the region of the genome that was within 0.5 Mb of the region on either side, truncated at chromosome ends. We removed bases within the flanking region that had mappability less than 0.5. We calculated a flanking mutation rate for each candidate region by dividing the number of mutations in our set of whole genomes that overlap valid flanking base positions by the number of valid bases within the flanking region. We calculated a raw mutation score (Equation 1) by dividing the rate (mutations per nucleotide) in the candidate region by the flanking mutation rate. We normalized this raw mutation score by subtracting the median score from all candidate regions and dividing each score by the median absolute deviation (mad) over all candidates (Equation 2). We initially planned to perform the normalization by flanking mutation rate separately for each tumour sample, but this was not feasible due to the sparsity of mutations in some samples. Equations for the raw and normalized recurrence scores are:

1 $$ raw\  score = \frac{\raisebox{1ex}{$T$}\!\left/ \!\raisebox{-1ex}{${T}_0$}\right.}{\raisebox{1ex}{$\left(L+R\right)$}\!\left/ \!\raisebox{-1ex}{$\left({L}_0+{R}_0\right)$}\right.} $$

Where T is the number of mutations observed in the target region, T_0_ is the length of the target region, L and R are the number of mutations in the left and right flanking regions of the target region, and L_0_ and R_0_ are the lengths of the left and right flanking regions.

2 $$ normalized\  score = \frac{raw\  score - median\left( raw\  score\right)}{mad\left( raw\  score\right)} $$

### Calculation of conservation score

For each candidate region, we also calculated a conservation score. Our strategy was to use a basepair level measure of conservation, and average across mutations to score a region based on conservation. We chose the PhyloP score [43] calculated on a 100-way species tree, which is available from the UCSC genome browser. PhyloP scores as implemented in the UCSC Genome Browser are negative log base 10 p-values for a likelihood ratio test against the null hypothesis of neutral evolution. The scores are positive when the test indicates that the nucleotide evolves more slowly (*i.e.* is conserved) and negative in the case that it evolves more quickly (acceleration). For each mutation, we mapped PhyloP scores of the base position at which the mutation occurred. Within each candidate region, we took the mean of the PhyloP scores for each mutation within the region as a raw conservation score. Similar to our recurrence score, we normalized this raw conservation score by subtracting the median score and dividing by the median absolute deviation.

### Calculation of combined score

For each candidate region, we calculated the combined score as the simple average of normalized recurrence and conservation scores.

### Statistical analysis

For comparison of scores in different classes of regions, we used Mann–Whitney tests, as implemented in R. we also performed simulations to compare the median scores of known driver regions to non-driver exonic regions. We repeated sampled with replacement 10,000 samples of non-driver regions with size equal to the number of candidate regions overlapping known driver regions, took the median score for each sample, and compared to the observed median for known driver genes.

### Collation of known driver genes

Driver genes were collated in humans by combining gene lists from two previously published lists of driver genes from Vogelstein *et al.* and Lawrence *et al.* [4, 6]. Gene names were taken from table S2A of Vogelstein *et al.* [4] and from Additional file 1: Table S2 from Lawrence *et al.* [6]. These gene names were entered into the UCSC Table Browser [39] to obtain hg19 coordinates for the coding exons of these genes, which were mapped to mutations using bedtools [41]. We considered a region to be a known driver if it overlapped a coding exon of a gene listed in either publication. In total, we constructed a set of 308 driver genes.

### Threshold sensitivity analysis

For all regions with greater than 2 mutations, we classified the region as either hypermutated or non-hypermuated based on whether the mutations per mutated sample in that region exceed a threshold, where exceeding the threshold resulted in classification as a hypermutated region. We classified regions in this way for thresholds of 1.1, 1.3, 1.3 and 1.5, and compared these classifications to a threshold of 1.2. For each comparison, we calculated the percent of regions that had the same classification (both hypermutated or both non-hypermutated) in the comparison.

### Transcription factor binding motif analysis

We obtained position weight matrices for human transcription factors using the “JASPAR2014” package in R [44], and searched for matches using the “searchSeq” function from the “TFBSTools” package [45] with default settings. We also selected recurrent mutations occurring within candidate regions and searched against the mutated sequence for transcription factors that matched the reference.

## Additional files

Additional file 1: Table S1. The number of samples with 1000 or more valid mutations included in our final analysis, as well as information about tumour type and original publication for each sample. For the ICGC samples we give ICGC project codes and use this to categorise tumour type throughout this work. Although some project codes imply the same tumour type (e.g. LICA-FR and LINC-JP are both liver cancers) we treat these separately in case these cohorts might have different properties, either technical or biological. **Table S2:** Top ten non-coding, non-hypermutated regions in terms of recurrence score within each cancer type. **Table S3:** Top ten non-coding, non-hypermutated regions in terms of combined score within each cancer type. (PDF 196 kb)
Additional file 2: Figure S1. Log10 of total mutations per genome, ordered by median mutations within each tumour type. **Figure S2:** For comparison, we show the location of mutations (black arrows) within a recurrent CTCF binding site that was highlighted in a previous analysis [28]. **Figure S3:** We show recurrence score (plotted as log(score + 2)) plotted against GC content. Regions with mutations per patient > 1.2 are in orange, with recurrence score > 10 and mutations per patient < = 1.2 in black, and all others in purple. **Figure S4:** Recurrent *TERT* promoter mutations identified in our data set. The mutations occur at one of the previously identified bases, generating a *de novo* ETS binding site. **Figure S5:** *PLEKHS1* recurrently mutated region that has previously been identified. We identify mutations at the same base position as previous analyses. **Figure S6:** UCSC browser image depicting a recurrently mutated region identified by our method. Mutations are depicted by black arrows. This region is flanked on the left by the gene *MED16.* **Figure S7:** Sequence logo depicting the *MEF2A* motif. Text above the logo is the reference sequence observed within the recurrently mutated region in the MED16 promoter. Mutated positions are depicted in red. **Figure S8:** UCSC browser image of a second recurrently mutated region identified by our method. Mutations are depicted by black arrows. **Figure S9:** Recurrently mutated region overlapping the miRNA *MIR142*. The region is highly conserved, as suggested by its inclusion among the top non-coding regions based on combined score. **Figure S10:** *MIR142* reference aligned with the sequence of mature microRNA has-miR142-5p. Mutated positions are depicted in red. **Figure S11:** Recurrently mutation overlapping an intron of the gene MSRA. The mutations occur primarily at two neighbouring bases. **Figure S12:** UCSC browser image of a recurrently mutated region overlapping an intron of the gene *PRIM2*. **Figure S13:** Sequence logo depicting the *FOXP2* motif. Text above the logo is the reference sequence observed within the recurrently mutated region in the *PRIM2* intron. Mutated positions are depicted in red. **Figure S14:** UCSC browser image depicting a recurrently mutated region in an intron of the DNA repair gene *RAD51B*. This region is mutated specifically in breast cancer. (PDF 4081 kb)
