# Supplementary material for: Identification of coding and non-coding mutational hotspots in cancer genomes
Source: BMC Genomics. 2017 Jan 5;18:17. doi: 10.1186/s12864-016-3420-9 (PMC5217664; doi:10.1186/s12864-016-3420-9)
Supplement: Additional file 1: Table S1. — The number of samples with 1000 or more valid mutations included in our final analysis, as well as information about tumour type and original publication for each sample. For the ICGC samples we give ICGC project codes and use this to categorise tumour type throughout this work. Although some project codes imply the same tumour type (e.g. LICA-FR and LINC-JP are both liver cancers) we treat these separately in case these cohorts might have different properties, either technical or biological. Table S2: Top ten non-coding, non-hypermutated regions in terms of recurrence score within each cancer type. Table S3: Top ten non-coding, non-hypermutated regions in terms of combined score within each cancer type. (PDF 196 kb) [file 12864_2016_3420_MOESM1_ESM.pdf]

Supplementary Tables 1-3 from "Identification of coding and non-coding mutational hotspots in cancer genomes" (Piraino and Furney).

**Source**	**Cancer type**	**Cancer cohort**	**Number of samples**
Alexandrov *et al.*	Acute lymphoblastic leukemia (ALL)	Acute lymphoblastic leukemia	1
ICGC	Bone	BOCA-FR	3
Alexandrov *et al.*	Breast	Breast	116
ICGC	Chronic lymphocytic leukemia (CLL)	Chronic lymphocytic leukemia	21
ICGC	Prostate	EOPC-DE	9
ICGC	Esophageal	ESAD-UK	97
Wang *et al.*	Gastric	Gastric	98
ICGC	Liver	LICA-FR	5
ICGC	Liver	LINC-JP	31
ICGC	Liver	LIRI-JP	238
Alexandrov et al.	Lung	Lung	24
ICGC	Lymphoma	MALY-DE	44
Alexandrov *et al.*	Medulloblastoma	Medulloblastoma	42
ICGC	Ovarian	OV-AU	75
ICGC	Pancreatic	PACA-AU	148
ICGC	Pancreatic	PACA-CA	151
ICGC	Pancreatic	PACA-IT	29
ICGC	Pancreatic	PAEN-AU	37
ICGC	Prostate	PRAD-CA	89
ICGC	Renal	RECA-EU	88
ICGC	Thyroid	THCA-SA	3

Supplementary Table 1: The number of samples with 1000 or more valid mutations included in our final analysis, as well as information about tumour type and original publication for each sample. For the ICGC samples we give ICGC project codes and use this to categorise tumour type throughout this work. Although some project codes imply the same tumour type (e.g. LICA-FR and LINC-JP are both liver cancers) we treat these separately in case these cohorts might have different properties, either technical or biological.

chr	start	End	mutated samples	score	Cohort	Annotation
chr10	75457700	75457750	4	1.4405	Breast	*AGAP5* intron
chr10	115511550	115511600	5	1.6216	Breast	*PLEKHS1* promoter
chr11	296200	296250	3	0.9419	Breast	Non-coding
chr14	69134600	69134650	3	1.2896	Breast	*RAD51B* intron
chr14	104675450	104675500	4	1.0002	Breast	Non-coding
chr16	88260400	88260450	3	0.9971	Breast	Non-coding
chr16	88463300	88463350	3	1.0136	Breast	Non-coding
chr17	5025550	5025600	3	1.1389	Breast	*ZNF232* intron
chr22	26714000	26714050	5	1.5020	Breast	*SEZ6L* intron
chr4	120322450	120322500	4	2.3094	Breast	Non-coding
chr1	52344450	52344500	4	27.1847	ESAD-UK	*NDR1* promoter
chr10	30966600	30966650	5	28.3009	ESAD-UK	Non-coding
chr11	63777600	63777650	4	27.7663	ESAD-UK	*MACROD1* intron
chr16	89081750	89081800	6	34.0315	ESAD-UK	Non-coding
chr17	521450	521500	5	34.1686	ESAD-UK	*VPS53* intron
chr17	552650	552700	6	42.0077	ESAD-UK	*VPS53* intron
chr17	78287250	78287300	6	47.7300	ESAD-UK	*RNF213* intron
chr19	49172650	49172700	4	27.2390	ESAD-UK	*NTN5* intron
chr7	151591850	151591900	5	33.9840	ESAD-UK	Non-coding
chr9	42858850	42858900	4	27.6646	ESAD-UK	LOC286297 ncRNA
chr1	46385450	46385500	6	4.1470	Gastric	*MAST2* intron
chr10	129059650	129059700	11	4.6119	Gastric	*DOCK1* intron
chr16	4039350	4039400	6	4.0935	Gastric	*ADCY9* intron
chr17	62640700	62640750	5	3.9717	Gastric	*SMURF2* intron
chr19	19088350	19088400	7	4.5256	Gastric	Non-coding
chr2	25089450	25089500	6	3.8879	Gastric	*ADCY3* intron
chr3	195892250	195892300	18	11.3319	Gastric	Non-coding
chr4	169312800	169312850	8	6.5470	Gastric	*DDX60L* intron
chr4	169313950	169314000	5	3.7960	Gastric	*DDX60L* intron
chr5	179868450	179868500	6	3.9871	Gastric	non-coding TF binding
chr10	89346150	89346200	5	9.4418	LIRI-JP	non-coding TF binding
chr16	1709700	1709750	4	11.7738	LIRI-JP	*CRAMP1L* intron
chr19	893450	893500	4	17.4535	LIRI-JP	*MED16* promoter
chr20	796800	796850	5	12.8574	LIRI-JP	CTCF binding
chr20	10151100	10151150	6	7.6185	LIRI-JP	*SNAL25-AS1* ncRNA
chr21	16350350	16350400	7	9.6832	LIRI-JP	*NRIP1* intron
chr3	43746350	43746400	7	10.0986	LIRI-JP	*ABHD5* intron
chr4	119394200	119394250	6	7.9062	LIRI-JP	Non-coding
chr4	142289250	142289300	7	7.3136	LIRI-JP	Non-coding
chr7	65090800	65090850	6	8.1183	LIRI-JP	Non-coding
chr1	169975450	169975500	3	-0.4557	OV-AU	*KIFAP3* intron
chr1	216051800	216051850	3	-0.4633	OV-AU	*USH2A* intron
chr12	88776700	88776750	3	0.0648	OV-AU	Non-coding
chr12	101545800	101545850	3	-0.1503	OV-AU	Non-coding
chr18	32342800	32342850	3	-0.2974	OV-AU	*DTNA* intron
chr2	69530800	69530850	3	1.0857	OV-AU	Non-coding
chr2	166337300	166337350	3	0.2182	OV-AU	*CSRNP3* intron
chr5	97091250	97091300	3	-0.0193	OV-AU	non-coding
chr5	131366350	131366400	3	0.3409	OV-AU	non-coding

chr8	117337100	117337150	4	0.3649	OV-AU	LINC00536 / TF binding
chr1	51589650	51589700	3	2.5931	PACA-AU	*C1orf185* intron
chr10	118803350	118803400	4	2.0360	PACA-AU	*KIAA15998* intron
chr14	74083000	74083050	3	3.4429	PACA-AU	non-coding
chr16	48483950	48484000	5	4.2544	PACA-AU	*MIR5095* non-coding
chr16	69602250	69602300	3	2.6323	PACA-AU	*NFAT5* intron
chr17	1600600	1600650	3	3.4882	PACA-AU	non-coding
chr2	191157850	191157900	3	2.0794	PACA-AU	*HIBCH* intron
chr2	203851600	203851650	3	2.2701	PACA-AU	non-coding
chr20	14680700	14680750	4	2.2964	PACA-AU	*MACROD2* intron
chr6	107807150	107807200	3	3.2244	PACA-AU	non-coding
chr1	21793400	21793450	3	4.0058	PACA-CA	*NBPF3* intron
chr1	27503450	27503500	3	4.3162	PACA-CA	non-coding
chr1	206519250	206519300	5	6.8580	PACA-CA	*SRGAP2* intron
chr10	89005600	89005650	3	3.5191	PACA-CA	*NUTM2A-AS1* ncRNA
chr15	67334650	67334700	4	4.1283	PACA-CA	non-coding TF binding
chr16	70039200	70039250	4	3.7022	PACA-CA	*PDXDC2P* ncRNA
chr5	141582500	141582550	4	4.5125	PACA-CA	non-coding
chr9	123950	124000	5	6.0043	PACA-CA	*CBWD1* intron
chr9	42540200	42540250	3	15.9414	PACA-CA	non-coding
chr9	70996500	70996550	4	3.9799	PACA-CA	*PGM5* intron
chr1	228032600	228032650	3	1.5023	PRAD-CA	*PRSS38* intron
chr15	41726800	41726850	3	1.4188	PRAD-CA	*RTF1* intron
chr15	73669700	73669750	4	2.2093	PRAD-CA	non-coding
chr16	173850	173900	3	1.7730	PRAD-CA	*NPRL3* intron
chr16	68244200	68244250	4	2.9728	PRAD-CA	*NFATC3* intron
chr19	49815100	49815150	3	1.3712	PRAD-CA	*SLC6A16* intron
chr2	131394050	131394100	4	2.2702	PRAD-CA	*POTEJ* intron
chr4	39684550	39684600	6	3.5422	PRAD-CA	non-coding
chr7	141323150	141323200	4	1.5960	PRAD-CA	*AGK* intron
chr9	66313600	66313650	3	2.4021	PRAD-CA	non-coding
chr1	46226150	46226200	6	2.0896	RECA-EU	non-coding
chr11	44336600	44336650	5	1.1582	RECA-EU	non-coding
chr12	655150	655200	6	1.3493	RECA-EU	*B4GALNT3* intron
chr12	122822000	122822050	5	1.4043	RECA-EU	*CLIP1* intron
chr2	84825300	84825350	7	1.3525	RECA-EU	*DNAH6* intron
chr3	46746150	46746200	6	3.1945	RECA-EU	*TMIE* intron
chr3	56204600	56204650	5	1.8347	RECA-EU	*ERC2* intron
chr6	57343100	57343150	12	4.5837	RECA-EU	*PRIM2* intron
chr7	28744950	28745000	6	1.6668	RECA-EU	*CREB5* intron
chr8	29901300	29901350	9	2.7300	RECA-EU	non-coding

Supplementary Table 2: Top ten non-coding, non-hypermutated regions in terms of recurrence score within each cancer type.

chr	start	End	mutated samples	score	Cohort	Annotation
chr10	75457700	75457750	4	0.5052	Breast	*APGAP5* intron
chr10	115511550	115511600	5	0.9404	Breast	*PLEKHS1* promoter
chr13	23615650	23615700	3	0.4879	Breast	non-coding
chr14	69134600	69134650	3	0.8344	Breast	*RAD51B* intron
chr16	10746650	10746700	4	0.5450	Breast	*TEXT5* intron / TF binding
chr16	88260400	88260450	3	0.6453	Breast	non-coding
chr16	88463300	88463350	3	0.6663	Breast	non-coding
chr19	42466450	42466500	3	0.9510	Breast	non-coding
chr4	120322450	120322500	4	0.6950	Breast	non-coding
chr6	168637600	168637650	3	0.9844	Breast	non-coding
chr1	52344450	52344500	4	13.6021	ESAD-UK	*NRD1* promoter
chr10	30966600	30966650	5	12.6044	ESAD-UK	non-coding
chr16	89081750	89081800	6	15.8425	ESAD-UK	non-coding
chr17	521450	521500	5	16.9202	ESAD-UK	*VPS53* intron
chr17	552650	552700	6	20.3809	ESAD-UK	*VPS53* intron
chr17	78287250	78287300	6	23.4776	ESAD-UK	*RNF213* intron
chr6	38461900	38461950	5	13.9464	ESAD-UK	*BTBD9* intron / CTCF binding
chr7	127898750	127898800	4	13.2956	ESAD-UK	non-coding
chr7	151591850	151591900	5	17.0298	ESAD-UK	non-coding
chr9	42858850	42858900	4	13.6816	ESAD-UK	LOC286297 ncRNA
chr11	31150650	31150700	3	2.2649	gastric	*DCDC1* intron
chr17	31038650	31038700	4	2.3389	gastric	*MYO1D* intron
chr17	59465950	59466000	3	4.6770	gastric	*BCAS3* intron
chr2	143949750	143949800	3	2.5988	gastric	*ARHGAP15* intron
chr3	195892250	195892300	18	5.8723	gastric	non-coding
chr4	169312800	169312850	8	3.6715	gastric	*DDX60L* intron
chr4	169313950	169314000	5	2.3140	gastric	*DDX60L* intron
chr6	43041350	43041400	5	2.3349	gastric	*KLC4* intron
chr6	50570100	50570150	3	2.8894	gastric	CTCF binding
chr8	65519150	65519200	3	2.7909	gastric	*CYP7B1* intron
chr16	1709700	1709750	4	5.8069	LIRI-JP	*CRAMP1* intron
chr16	52531300	52531350	3	9.9815	LIRI-JP	*TOX3* intron
chr19	893450	893500	4	11.9033	LIRI-JP	*MED16* promoter
chr2	7342150	7342200	3	7.7298	LIRI-JP	non-coding
chr2	60684450	60684500	3	5.9503	LIRI-JP	*BCL11A*
chr20	796800	796850	5	6.1129	LIRI-JP	CTCF binding
chr21	16350350	16350400	7	5.6199	LIRI-JP	*NRIP1* intron
chr3	43746350	43746400	7	5.4083	LIRI-JP	*ABHD5* intron
chr3	187439750	187439800	3	8.5100	LIRI-JP	*BCL6* intron
chr9	36940450	36940500	3	5.2957	LIRI-JP	*PAX5* intron
chr1	169975450	169975500	3	-0.0932	OV-AU	*KIFAP3* intron
chr1	216051800	216051850	3	-0.2351	OV-AU	*USH2A* intron
chr12	88776700	88776750	3	0.0358	OV-AU	non-coding
chr12	101545800	101545850	3	-0.0795	OV-AU	non-coding
chr18	32342800	32342850	3	-0.0308	OV-AU	*DTNA* intron
chr2	69530800	69530850	3	0.0374	OV-AU	non-coding

chr2	166337300	166337350	3	0.0700	OV-AU	*CSRNP3* intron
chr5	97091250	97091300	3	-0.3319	OV-AU	non-coding
chr5	131366350	131366400	3	0.2291	OV-AU	non-coding
chr8	117337100	117337150	4	0.6292	OV-AU	LINC00536 ncRNA / TF binding
chr14	74083000	74083050	3	0.9026	PACA-AU	non-coding
chr16	48483950	48484000	5	2.1310	PACA-AU	*MIR5059* ncRNA
chr16	69602250	69602300	3	1.9837	PACA-AU	*NFAT5* intron
chr17	1600600	1600650	3	1.7198	PACA-AU	non-coding
chr19	5250450	5250500	3	1.4586	PACA-AU	*PTPRS* intron
chr2	203851600	203851650	3	1.7673	PACA-AU	non-coding
chr20	14680700	14680750	4	0.9078	PACA-AU	*MACROD2* intron
chr6	107807150	107807200	3	1.7200	PACA-AU	non-coding
chr7	122981650	122981700	3	0.9430	PACA-AU	non-coding
chr8	120286150	120286200	3	0.9145	PACA-AU	non-coding
chr1	21793400	21793450	3	2.0401	PACA-CA	*NBPF3* intron
chr1	27503450	27503500	3	4.8274	PACA-CA	non-coding
chr1	206519250	206519300	5	2.8859	PACA-CA	*SRGAP2* intron
chr10	89005600	89005650	3	2.0901	PACA-CA	*NUTM2A-AS1* ncRNA
chr18	44002100	44002150	3	2.0086	PACA-CA	*RNF165* intron
chr5	99390600	99390650	3	3.9516	PACA-CA	non-coding
chr9	123950	124000	5	3.3592	PACA-CA	*CBWD1* intron
chr9	35357550	35357600	3	1.8179	PACA-CA	*UNC13B* intron
chr9	42540200	42540250	3	8.0164	PACA-CA	non-coding
chr9	70996500	70996550	4	1.8112	PACA-CA	*PGM5* intron
chr10	16330150	16330200	3	0.4259	PRAD-CA	non-coding
chr14	75148450	75148500	3	0.5223	PRAD-CA	*AREL1* intron
chr15	73669700	73669750	4	1.0125	PRAD-CA	non-coding
chr16	173850	173900	3	0.9023	PRAD-CA	*NPRL3* intron
chr16	68244200	68244250	4	0.3793	PRAD-CA	*NFATC3* intron
chr17	29476300	29476350	3	0.5816	PRAD-CA	*NF1* intron
chr19	7152400	7152450	3	0.4746	PRAD-CA	*INSR* intrron
chr2	203520450	203520500	3	0.4251	PRAD-CA	*FAM117B* intron
chr4	39684550	39684600	6	1.0123	PRAD-CA	non-coding
chr7	143666450	143666500	3	0.6287	PRAD-CA	non-coding
chr1	46226150	46226200	6	1.2094	RECA-EU	non-coding
chr12	69755800	69755850	4	1.0794	RECA-EU	*YEATS4* intron
chr12	122822000	122822050	5	1.0534	RECA-EU	*CLIP1* intron
chr16	18820800	18820850	4	3.3245	RECA-EU	*SMG1* UTR
chr18	57125350	57125400	4	1.0331	RECA-EU	*CCBE1* intron
chr3	46746150	46746200	6	2.4872	RECA-EU	*TMIE* intron
chr3	56204600	56204650	5	1.2752	RECA-EU	*ERC2* intron
chr6	57343100	57343150	12	2.0394	RECA-EU	*PRIM2* intron
chr7	28744950	28745000	6	1.0070	RECA-EU	*CREB5* intron
chr8	29901300	29901350	9	1.5436	RECA-EU	non-coding

Supplementary Table 3: Top ten non-coding, non-hypermutated regions in terms of combined score within each cancer type.
