# Supplementary material for: Identification of coding and non-coding mutational hotspots in cancer genomes
Source: BMC Genomics. 2017 Jan 5;18:17. doi: 10.1186/s12864-016-3420-9 (PMC5217664; doi:10.1186/s12864-016-3420-9)
Supplement: Additional file 2: Figure S1. — Log10 of total mutations per genome, ordered by median mutations within each tumour type. Figure S2: For comparison, we show the location of mutations (black arrows) within a recurrent CTCF binding site that was highlighted in a previous analysis [28]. Figure S3: We show recurrence score (plotted as log(score + 2)) plotted against GC content. Regions with mutations per patient > 1.2 are in orange, with recurrence score > 10 and mutations per patient < = 1.2 in black, and all others in purple. Figure S4: Recurrent TERT promoter mutations identified in our data set. The mutations occur at one of the previously identified bases, generating a de novo ETS binding site. Figure S5: PLEKHS1 recurrently mutated region that has previously been identified. We identify mutations at the same base position as previous analyses. Figure S6: UCSC browser image depicting a recurrently mutated region identified by our method. Mutations are depicted by black arrows. This region is flanked on the left by the gene MED16. Figure S7: Sequence logo depicting the MEF2A motif. Text above the logo is the reference sequence observed within the recurrently mutated region in the MED16 promoter. Mutated positions are depicted in red. Figure S8: UCSC browser image of a second recurrently mutated region identified by our method. Mutations are depicted by black arrows. Figure S9: Recurrently mutated region overlapping the miRNA MIR142. The region is highly conserved, as suggested by its inclusion among the top non-coding regions based on combined score. Figure S10: MIR142 reference aligned with the sequence of mature microRNA has-miR142-5p. Mutated positions are depicted in red. Figure S11: Recurrently mutation overlapping an intron of the gene MSRA. The mutations occur primarily at two neighbouring bases. Figure S12: UCSC browser image of a recurrently mutated region overlapping an intron of the gene PRIM2. Figure S13: Sequence logo depicting the FOXP2 motif. Text above the logo is the reference seq [file 12864_2016_3420_MOESM2_ESM.pdf]

Supplementary Figures 1-11 from "Identification of coding and non-coding mutational hotspots in cancer genomes" (Piraino and Furney).

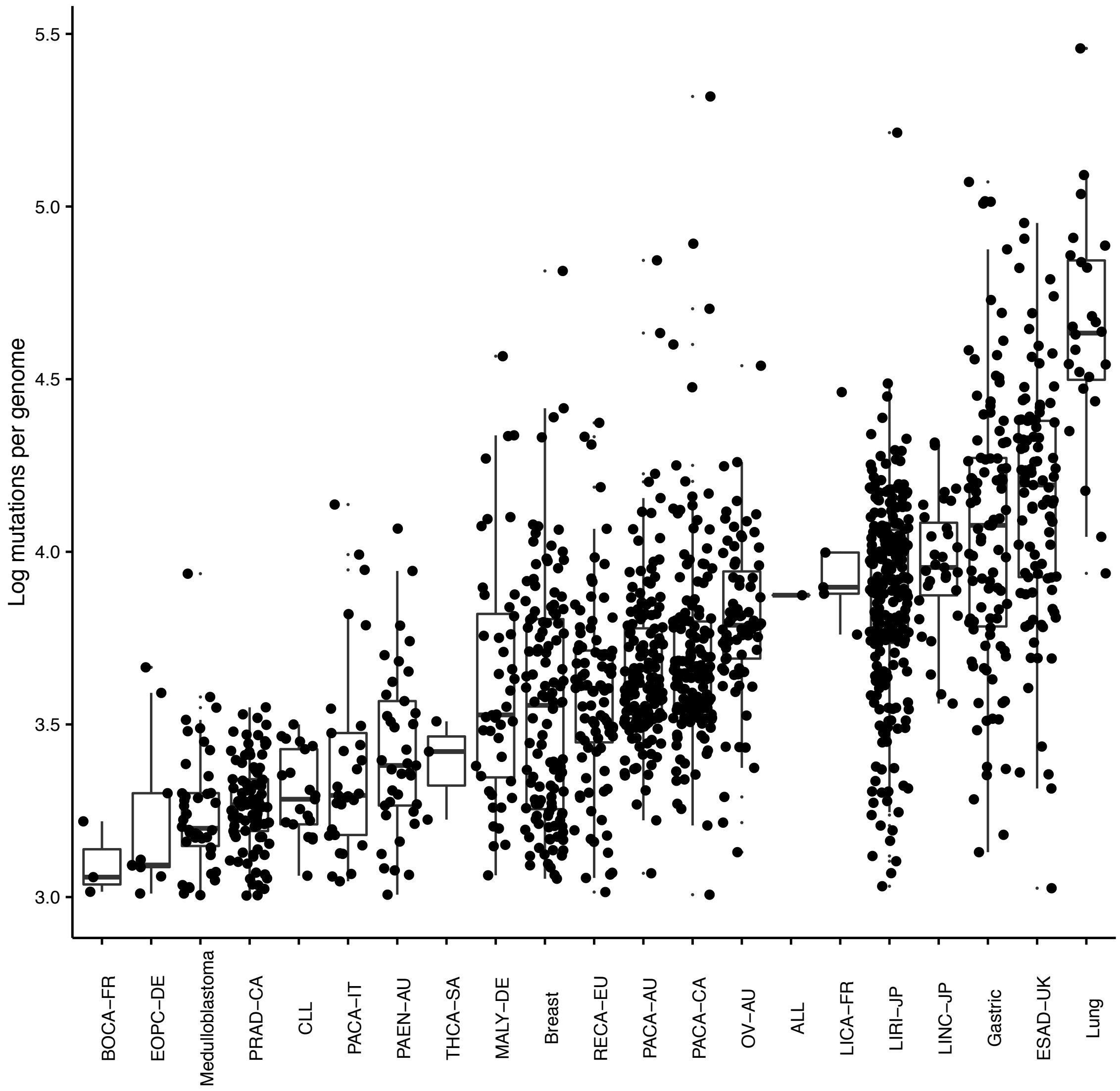

Supplementary Figure 1: Log10 of total mutations per genome, ordered by median mutations within each tumour type. There is considerable variation both within and between cancer types. The most highly mutated cancer types are generally associated with intense exposure to known mutational process, such as tobacco smoke in lung cancer and DNA repair defects in gastric cancer.

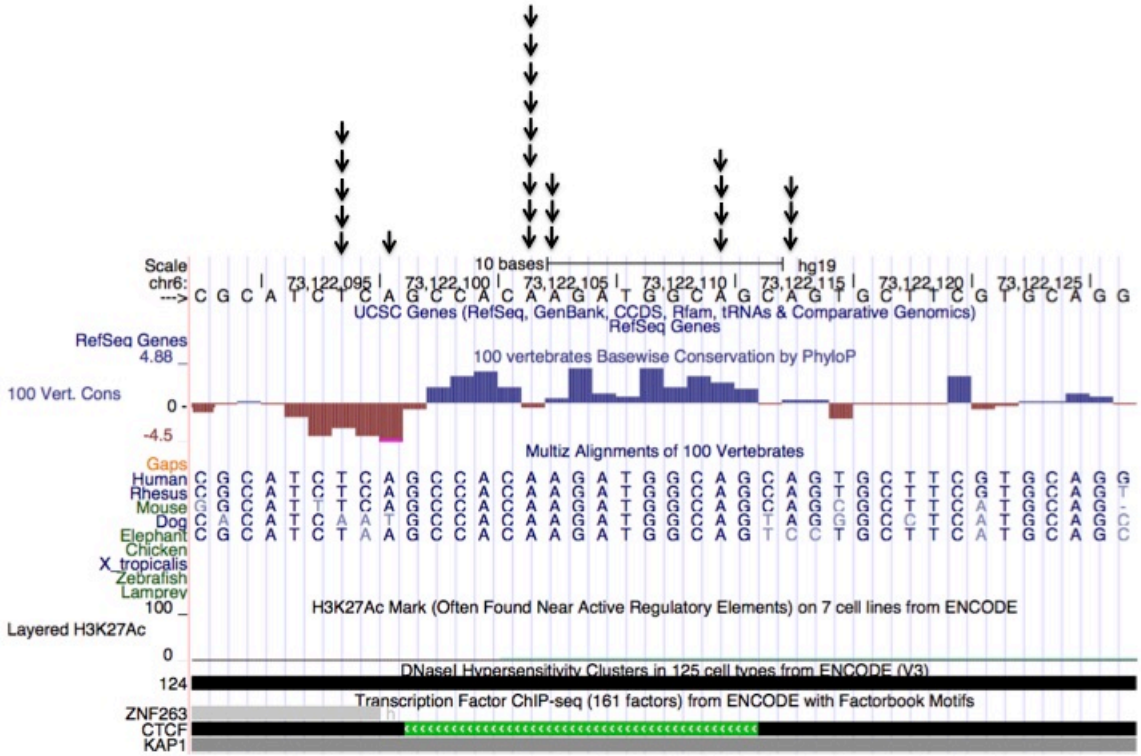

Supplementary Figure 2: For comparison, we show the location of mutations (black arrows) within a recurrent CTCF binding site that was highlighted in a previous analysis [28]. The mutation positions and nucleotide changes observed in our sample match those identified in this previous analysis, despite the fact that our dataset lacks any colorectal cancer samples.

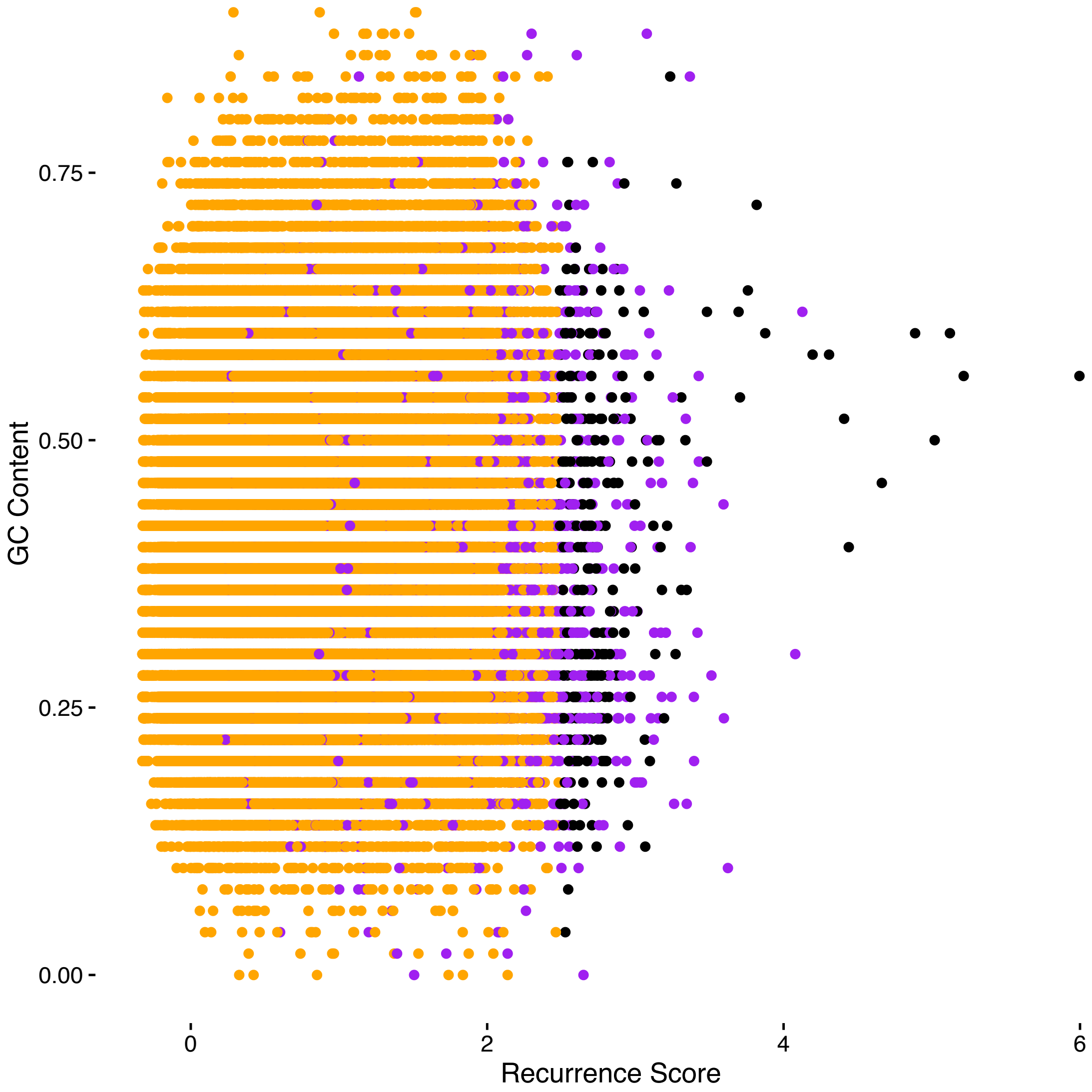

Supplementary Figure 3: We show recurrence score (plotted as log(score + 2)) plotted against GC content. Regions with mutations per patient > 1.2 are in orange, with recurrence score > 10 and mutations per patient <= 1.2 in black, and all others in purple.

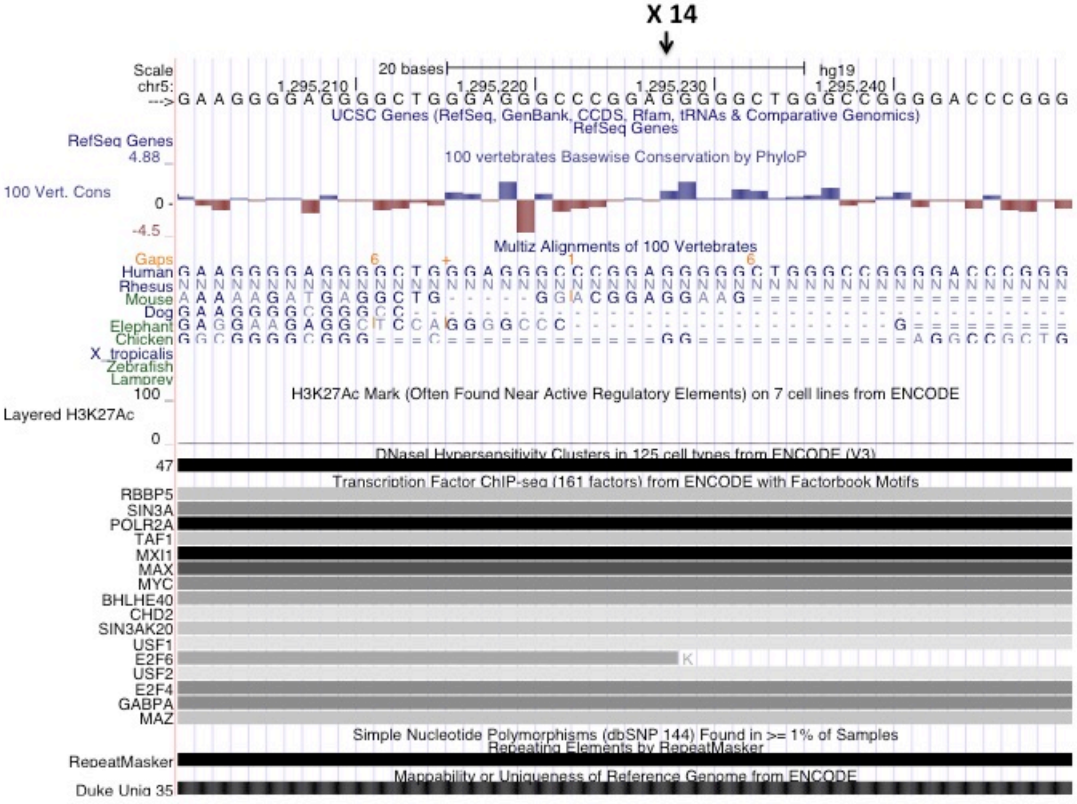

Supplementary Figure 4: Recurrent *TERT* promoter mutations identified in our data set. The mutations occur at one of the previously identified bases, generating a *de novo* ETS binding site.

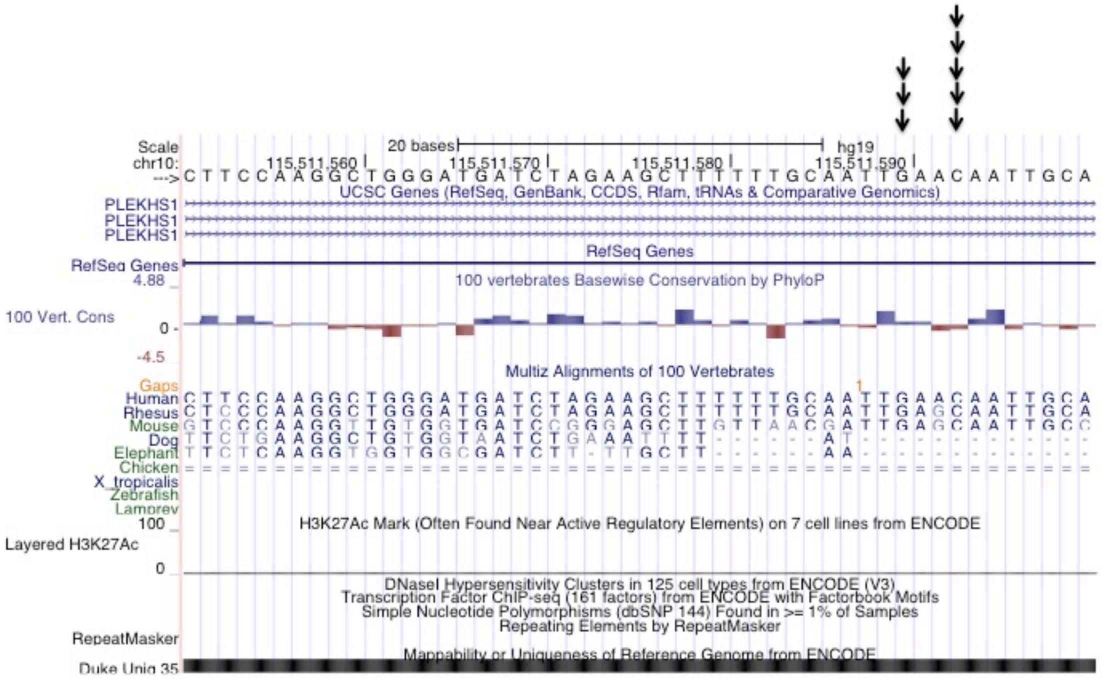

Supplementary Figure 5: *PLEKHS1* recurrently mutated region that has previously been identified. We identify mutations at the same base position as previous analyses.

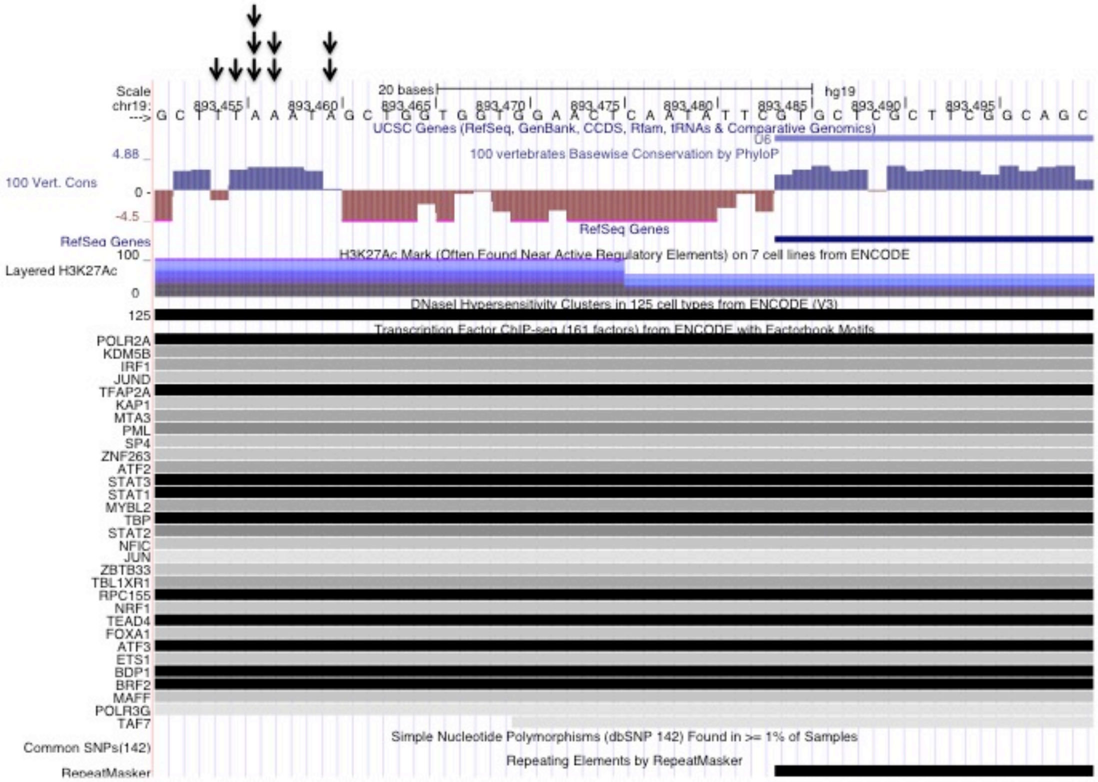

Supplementary Figure 6: UCSC browser image depicting a recurrently mutated region identified by our method. Mutations are depicted by black arrows. This region is flanked on the left by the gene *MED16.* The mutations observed in this region are focused within a conserved region overlapping a region of the genome with ENCODE evidence of transcription factor binding, possibly indicating selection.

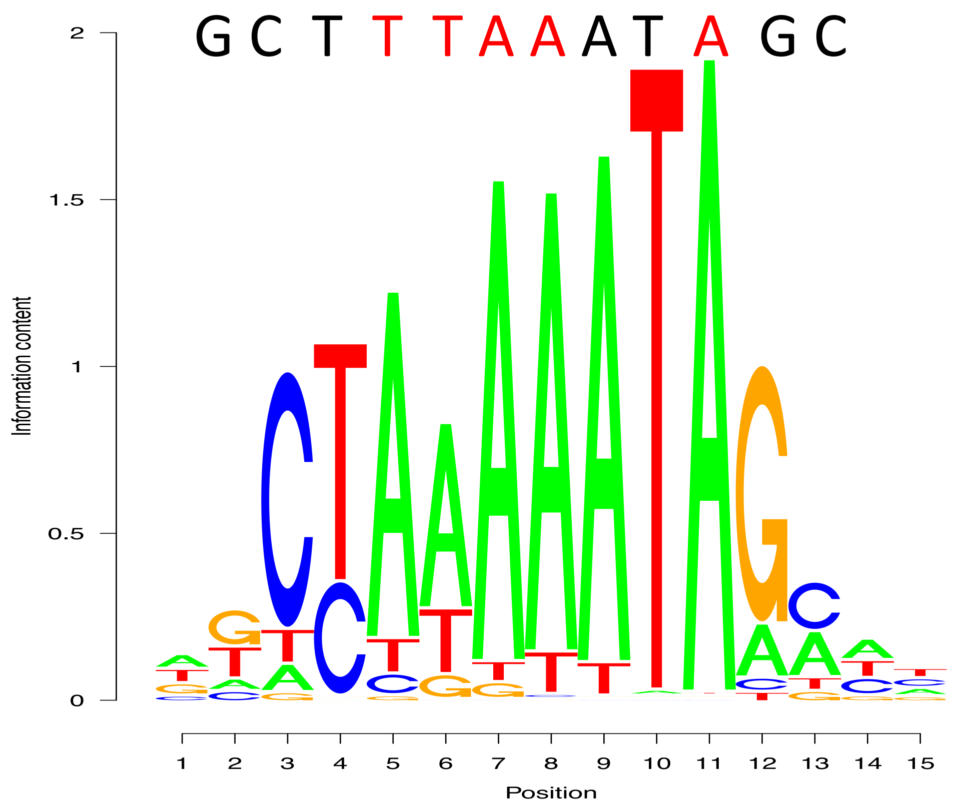

Supplementary Figure 7: Sequence logo depicting the *MEF2A* motif. Text above the logo is the reference sequence observed within the recurrently mutated region in the MED16 promoter. Mutated positions are depicted in red.

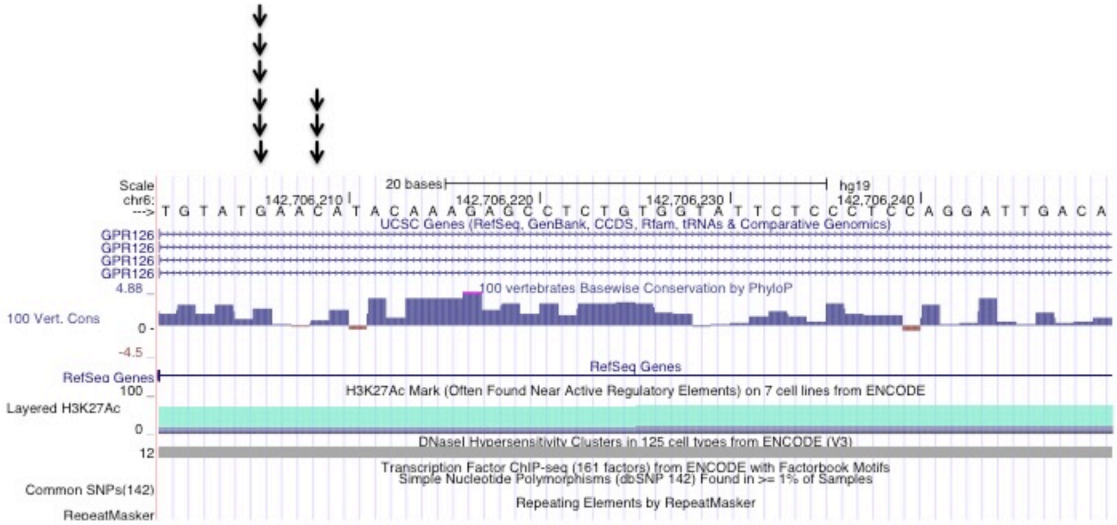

Supplementary Figure 8: UCSC browser image of a second recurrently mutated region identified by our method. Mutations are depicted by black arrows. This region overlaps a reasonably conserved intron of the gene *GPR126.* The mutations within this region occur exclusively at two nucleotides in a wholly mutually exclusive manner.

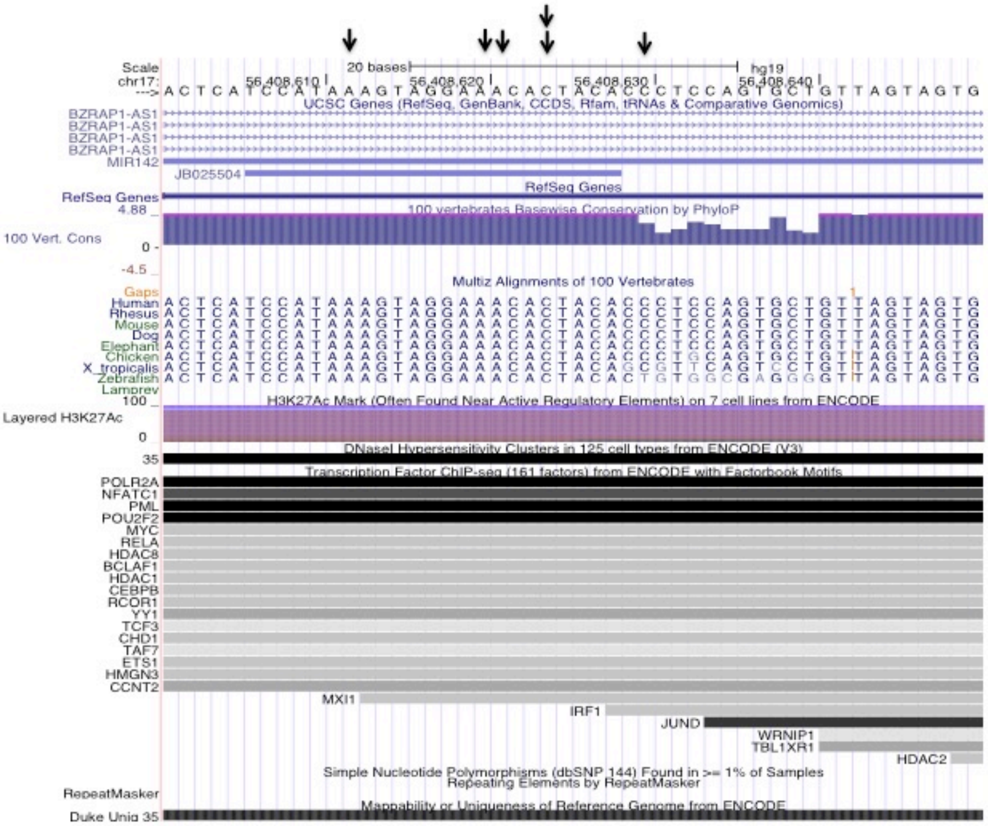

Supplementary Figure 9: Recurrently mutated region overlapping the miRNA *MIR142*. The region is highly conserved, as suggested by its inclusion among the top non-coding regions based on combined score. The mutations are spread throughout the region and occur exclusively in lymphoma samples, suggesting that this region may be a target of somatic hypermutation, not necessarily selection.

50bp window: CACTCATCCATAAAGTAGGAAACACTACACCCTCCAGTGCTGTTAGTAGTG

hsa-miR-142-5p: CATAAAGTAGAAAGCACTACT

Supplementary Figure 10: *MIR142* reference aligned with the sequence of mature microRNA has-miR142-5p. Mutated positions are depicted in red.

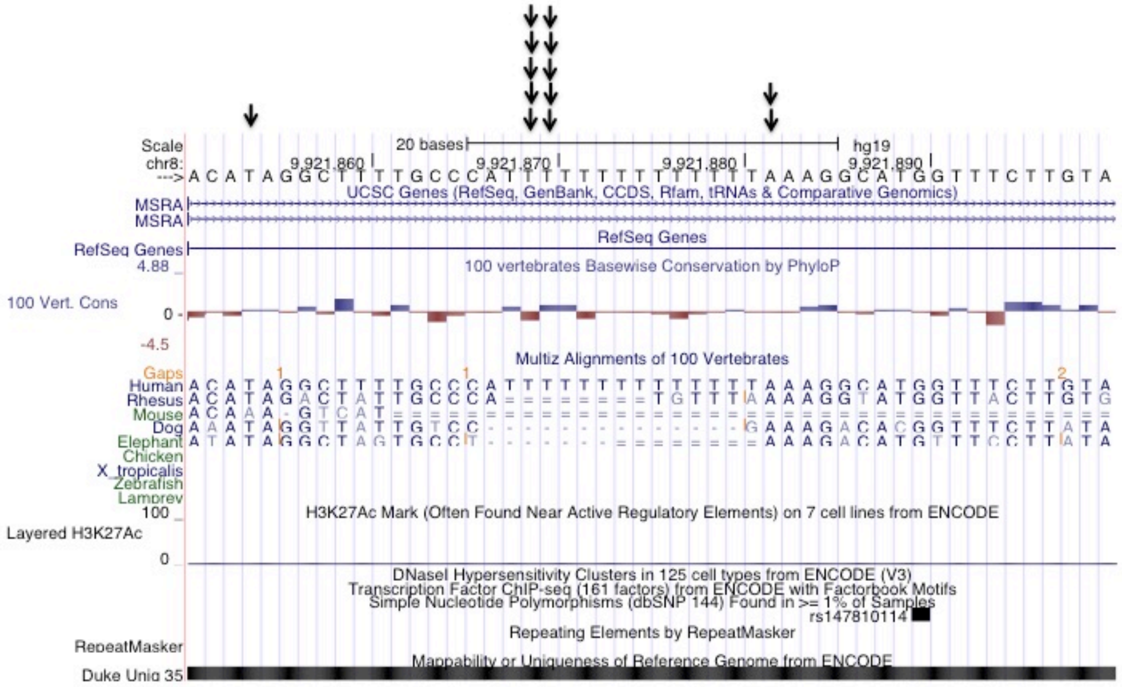

Supplementary Figure 11: Recurrently mutation overlapping an intron of the gene MSRA. The mutations occur primarily at two neighbouring bases.

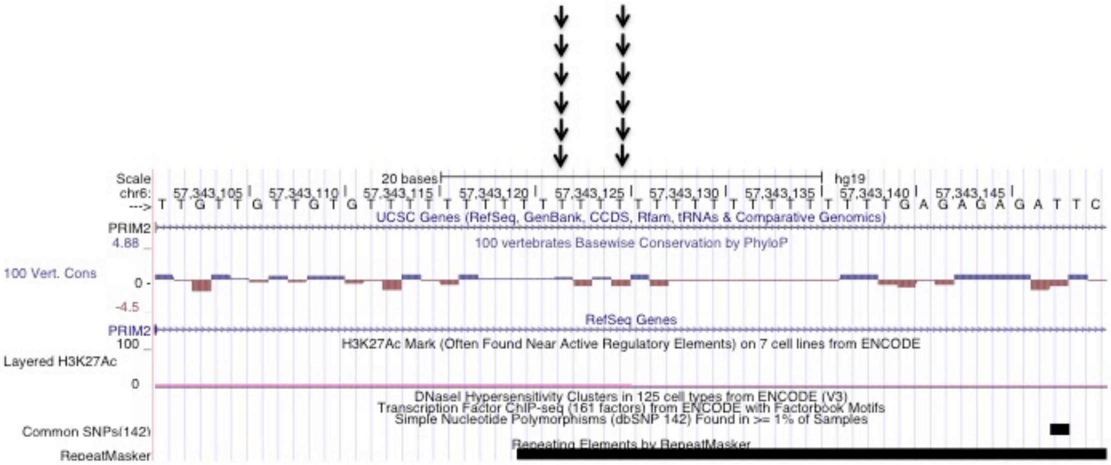

Supplementary Figure 12: UCSC browser image of a recurrently mutated region overlapping an intron of the gene *PRIM2*. Mutations are depicted as black arrows. All mutations within the region occur at one of two nucleotides and are mutually exclusive.

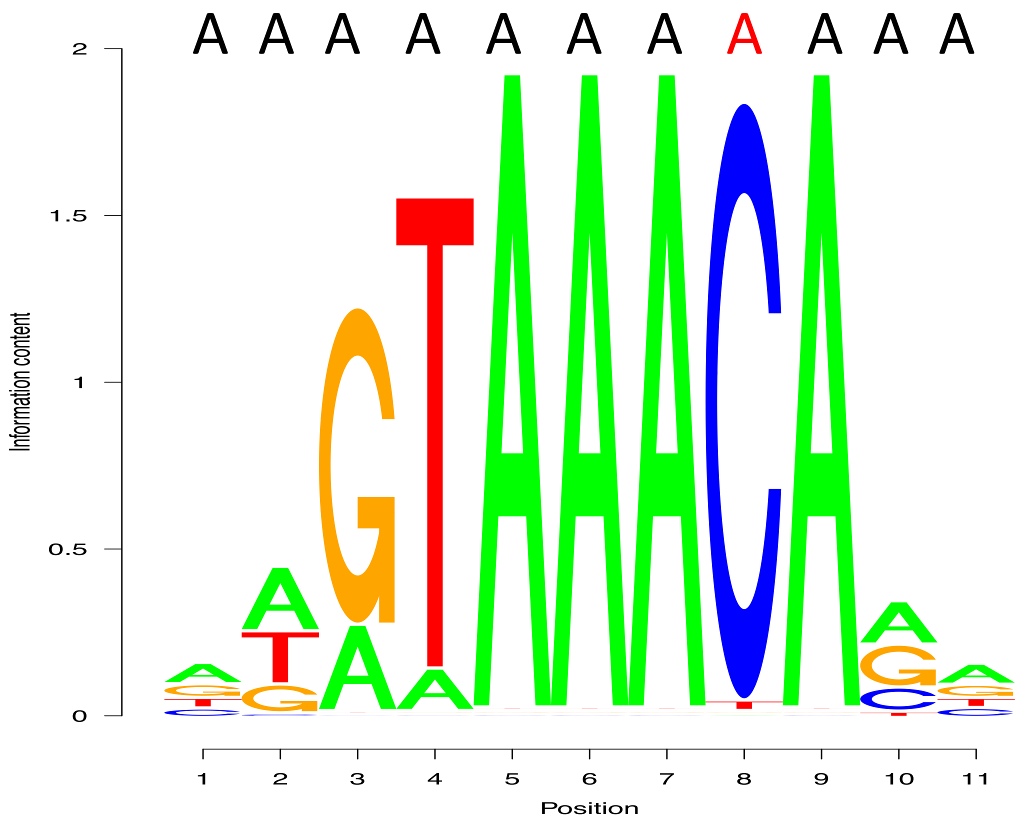

Supplementary Figure 13: Sequence logo depicting the *FOXP2* motif. Text above the logo is the reference sequence observed within the recurrently mutated region in the *PRIM2* intron. Mutated positions are depicted in red.

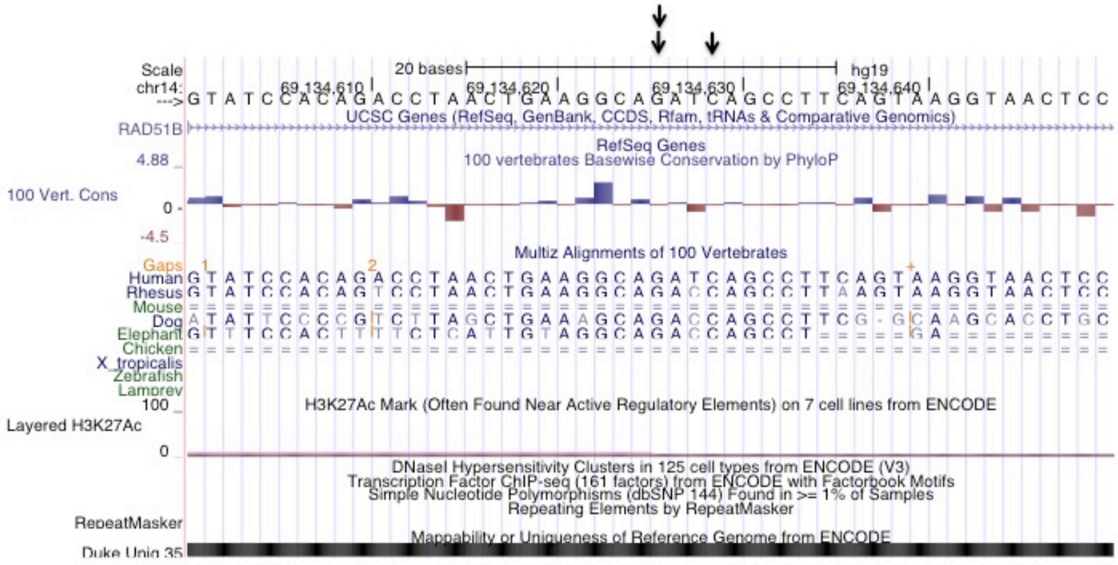

Supplementary Figure 14: UCSC browser image depicting a recurrently mutated region in an intron of the DNA repair gene *RAD51B*. This region is mutated specifically in breast cancer.
